# Host Range Breadth Correlates with Genic Diversity in Honeybee Phages

**DOI:** 10.1093/gbe/evag152

**Published:** 2026-07-03

**Authors:** Chris R P Robinson, Adam G Dolezal, Ivan Liachko, Irene L G Newton

**Affiliations:** Department of Biology, Indiana University Bloomington, Bloomington, IN 47405, USA; Department of Entomology, University of Illinois at Urbana-Champaign, Urbana, IL 61801, USA; Phase Genomics, Seattle, WA, USA; Department of Biology, Indiana University Bloomington, Bloomington, IN 47405, USA

**Keywords:** Bacteriophage ecology, Bacteriophage evolution, Population genetics, Honeybee microbiome, Metagenomic hiC

## Abstract

Bacteriophages can evolve rapidly. Mutation and recombination via horizontal gene transfer allow them to counter adaptive responses by microbial hosts. However, little is known about the genomic processes underlying phage evolution within an ecological context—especially within natural microbial communities. This is due in part to the difficulty in resolving aspects of phage ecology, such as host range. To better understand the interplay of phage ecology and evolution within natural microbial communities, we combined measures of phage host range in vivo with measures of genome evolution in order to infer the evolutionary pressures acting on phage genomes within individual honeybee worker microbiomes. We show that near-identical phage genomes, cooccurring across multiple honeybee colonies, exhibit large variation with respect to gene modules, despite retaining a highly similar core genome. Estimates of genic diversity suggest deviations from neutral evolutionary models and identify loci under putative diversifying selection. We then use HiC-resolved metagenomics and show that the honeybee gut contains a dense phage community that exhibits a wide degree of host range variation. This variation differed across individual metagenomes in both the number and phylogenetic distance of potential hosts. We show that common measures of genetic variation positively correlate with host range in bee-associated phages and that functional targets of diversifying selection are partitioned differently between broad or narrow host range phages. Our work underscores the high host range variation associated with phages within host-associated microbial communities and provides evidence that this variation impacts rates of phage evolution.

Significance It has been increasingly observed that natural populations of bacteriophages exhibit wide variation in host range, which calls into question our understanding regarding the coevolutionary dynamics between bacteria and their phage predators. Combining metagenomic HiC with the model honeybee worker microbiome, we quantify phage host range variation in vivo among isolated, conspecific phage populations and show that broader host ranges are associated with higher rates of genomic variation. We infer signatures of adaptive evolution and identify that the targets of diversifying selection differ among broad range and narrow range phages. These results underscore the ecological relevance of phage host range within host-associated microbial communities and highlights the genomic variation associated with phage ecological diversity.

## Introduction

The microbiome of the honeybee worker comprises myriad entities, including bacteria, microbial eukaryotes, and phages. Within this system, the taxonomic composition of the bacterial community has been extensively studied (Moran et al. 2012; Sabree et al. 2012) and has been shown to be important for nutrient provisioning (Parish et al. 2022), protection from pathogens (Miller et al. 2021), and metabolite detoxification (Zheng et al. 2016). Although there have been a few key metagenomic studies (Engel and Moran 2013; Regan et al. 2018; Shell and Rehan 2022; Caesar et al. 2024), widespread sequencing of the honeybee microbiome has been carried out mostly via 16S rRNA gene amplicon sequencing, which is limited in its ability to investigate functional diversity within bacterial genera and species. Further, the use of amplicon sequencing overlooks important drivers of microbial evolution and ecology, such as mobile genetic elements (MGEs, ie plasmids and bacteriophages). In contrast to our understanding of the microbial community within the honeybee worker, our knowledge of both the ecology and evolution of honeybee-associated phage communities is nascent.

Phages are among the most important entities within microbial communities due to their symbiotic interactions with microbial hosts as well as their ability to mediate conspecific and interspecific horizontal gene transmission. However, phages are almost always embedded within dense microbial communities and exhibit high variation in transmission rate (Sheppard et al. 2021) and gene content (Orlek et al. 2023). To control for this extensive variability in naturally occurring phages, most microbe-phage interactions have been studied in homogenous, liquid cultures (Koskella and Meaden 2013). These environments limit evolutionary and ecological processes that might select for phage variation, such as coevolution with multiple bacterial strains or recombination among phage genetic backgrounds. Though these controlled approaches have helped us develop a fundamental understanding of microbe-phage coevolution, they are limited in helping us to understand the complex factors that influence phage-encoded trait variability. The ecological and evolutionary impact of phages within natural microbial communities is largely understudied. For example, phages are hypothesized to drive changes in microbial community composition (Winter et al. 2010; Breitbart 2012; de Jonge et al. 2019; Flynn et al. 2022; Liao et al. 2023), but evidence for this is inconsistent (Schwalbach et al. 2004; Weinbauer et al. 2006; Mumford and Friman 2017; Zhang et al. 2023).

Recent work in *E. coli* and *Vibrio* systems has shown that phages exhibit wide variation in host range diversity, with phages partitioning into specialist, generalist, or “multiscale” networks (Piel et al. 2022; Borin et al. 2023). However, nearly all studies of phage evolution in natural systems (including the mentioned studies) have focused their efforts on a focal phage-bacterial pair or a single bacterial species complex. The large amount of diversity present within phage populations and the relatively few mutations necessary for large host range shifts (eg shifting across genera and families) suggest that phage host range variation (Ando et al. 2015) might be broader than previously thought.

Contemporary studies across several natural systems (Peters et al. 2015; de Jonge et al. 2019; Göller et al. 2021; George and Hug 2023; Hwang et al. 2023) have shown that phages likely associate with several phylogenetically distant microbial hosts. The stability and frequency of these interactions are thought to be mediated by host population diversity, community diversity, and transmission rate (May 1991; Janz et al. 2006; Rankin et al. 2011; Kamiya et al. 2014; Johnson et al. 2016). In turn, variation at the level of phage transmission rate has been shown to affect the evolution of phage virulence (Wendling et al. 2022) as well as having important consequences on phage ecology and stability (Huisman et al. 2025). Our understanding of whether and how this variation might impact measures of genic diversity in phage genomes remains an open question. Empirical and theoretical data from myriad systems, including bacterial symbionts, support hypotheses that predict a positive correlation between transmission rate and genetic diversity (Dobler and Farrell 1999; Lawton et al. 2011; Li et al. 2014; Russell et al. 2017; Eck et al. 2019; Robinson et al. 2024). A powerful model system with which to explore phage dynamics within host-associated microbial communities is the western honeybee (*Apis mellifera*) due to their long coevolutionary history with their gut microbiome (Engel et al. 2012), which has resulted in a stable, relatively low complexity microbial community. A handful of studies have explored bee-associated phages (Bonilla-Rosso et al. 2020; Deboutte et al. 2020; Busby et al. 2022; Bueren et al. 2023; Caesar et al. 2024; Sbardellati and Vannette 2024; Ndiaye et al. 2025), and these studies suggest that bees are associated with a diverse phage community that exhibits high variability in phage population sizes and rates of coevolution with microbial hosts. CRISPR-based methods predict that *Gilliamella*, *Lactobacillus*, *Bifidobacterium*, and *Bombilactobacillus* have historically been frequent hosts for phages (Bonilla-Rosso et al. 2020; Deboutte et al. 2020; Busby et al. 2022; Ndiaye et al. 2025). However, as not all bee-associated bacteria encode CRISPR systems, these assays may be limited by *in silico* approaches. More traditional approaches, incorporating phage isolation and plaque formation, have only been observed in *Bifidobacterium*, though we note these approaches also suffer from significant biases and limitations (Bonilla-Rosso et al. 2020; Ndiaye et al. 2025).

Here, we sought to better understand phage genome evolution within the ecological context of the honeybee worker gut microbiome. As honeybees have long coevolved with a core set of microbial taxa, and these taxa have been observed to engage in syntrophic interactions, we hypothesized that phages with variable host ranges will be prevalent within honeybee worker microbiomes (Moran et al. 2012; Kwong et al. 2014). Phages with variable host ranges have been observed in myriad natural systems (Hwang et al. 2023), and the combination of spatial heterogeneity within bee guts and the frequent horizontal transmission between individual bees are likely to result in phages with generalist ecological strategies (Russell et al. 2017). We further hypothesized that a subset of phages will stably cooccur across multiple honeybee colonies—despite potentially high bacterial strain variation—due to the stability and consistency of the honeybee worker gut community. Following these hypotheses, we predict that increasing phage host range will be associated with an increase in observed intrapopulation genetic diversity as a consequence of either coevolutionary (such as diversifying selection on genes associated with microbial-phage interactions) or demographic processes (such as recent population expansion as phages encounter new hosts). To test these hypotheses, we conducted deep metagenomic and HiC-proximity ligation sequencing of three age-matched honeybee worker pools, each originating from one of three colonies governed by a single-drone-inseminated (SDI) queen and held in the same apiary. We show that colonies harbor similar bacterial communities, as expected, but in contrast to our expectations, phage communities are highly heterogeneous and fairly specific to each colony. We identify a subset of near-identical phages that cooccur between multiple honeybee colonies and show that near-identical phage genomes exhibit a combination of highly variable genomic regions in addition to a core genome of high shared identity. By examining HiC networks, we show that phage host range is dynamic within honeybee worker microbiomes and that phage conspecifics exhibit wide variation in the number and phylogenetic diversity of their microbial hosts. Finally, we show that increasing phage host range positively correlates with measures of genic variation. Using the honeybee model to study natural microbial-phage dynamics in vivo, our results find that the gut microbiome contains a highly diverse phage community that exhibits a wide range of host range variation among bacterial hosts; this variation may affect both microbial and phage evolution in a natural, animal-associated microbial community.

## Results

### Metagenomic Assembly Recovers Core Microbial Taxa and cooccurring Phage Populations

We performed metagenomic and Hi-C sequencing of three pools of honeybee workers. Each pool consisted of dissected guts from 15 age-matched workers, collected from one of three healthy, SDI colonies, housed at the same apiary (the Bee Research Facility at University of Illinois Champaign-Urbana). Microbes were enriched through differential centrifugation from these pools and used for gDNA extraction (see Methods). Previous work has quantified 16S rRNA gene-based abundance within this sample set (Robinson et al. 2025). We assembled and binned microbial MAGs from each of the metagenomes by combining data from multiple binning strategies and from information gleaned from HiC-inferred contig-to-contig linkages (see Methods). Overall, we recovered 28 genomes from our binning strategy and which are representative of all core honeybee genera via inference by 16S rRNA gene analysis. These 28 genomes include 4 *Apilactobacillus*, 3 *Bartonella*, 5 *Bombilactobacillus*, 1 *Enterobacter*, 2 *Bifidobacterium*, 1 *Frischella*, 3 *Lactobacillus*, 3 *Commensalibacter*, 3 *Snodgrassella*, and 3 *Gilliamella*. However, only 13 of these genomes met the completeness (≥50%) and contamination (≤10%) thresholds based on the Genomic Standards Consortium (Bowers et al. 2017). The 13 microbial genomes that passed our quality thresholds were dereplicated at 97% ANI and 85% breadth, resulting in 9 representative genomes which were used for read recruitment in each of the individual metagenomes. CheckM quality scores are provided for all 28 recovered MAGs (see Table S1).

These nine microbial metagenomically assembled genomes (mMAGs) are representative of four core phylotypes (*Gilliamella apicola*, *Snodgrassella alvi*, *Bombilactobacillus mellifer*, and *Bombilactobacillus mellis*) and five noncore phylotypes (*Commensalibacter*, *Frischella*, *Enterobacter*, *Apilactobacillus*, and *Bartonella*) of the honeybee worker microbiome. To clarify, we use “core phylotypes” here as reference to the frequently observed taxa seen in honeybee microbiomes (Martinson et al. 2011). The average genome-wide coverage of these high-quality mMAGs was comparable across metagenomes, with the exception of *Enterobacter* which was more abundant in Metagenome C (Table S2). The recovered mMAGs represent the majority of observed phylogenetic diversity within the honeybee worker gut microbiome (Moran et al. 2012), though we note that the removal of contaminated *Lactobacillus* and *Bifidobacterium* genome assembles limits our ability to observe MGE interactions across all core microbial taxa within the honeybee gut. However, these MAGs were associated with lower abundance in our samples as *Lactobacillus* accounted for 5.39%, 2.27%, and 9.56% of 16S rRNA gene abundance in Metagenomes A, B, and C while *Bifidobacterium* 16S rRNA gene abundance accounted for only 1.43%, <1%, and 3.26% in Metagenomes A, B, and C, respectively (Fig. S1). The high contamination scores (see Table S1) associated with *Bifidobacterium* and *Lactobacillus* assemblies suggest that a combination of low coverage and high strain diversity complicated attempts to recover high-quality microbial bins in these taxa. With the recovery of core microbial taxa and known phage hosts (Busby et al. 2022), we next characterized the bacteriophage diversity within our samples. After assembly, we recovered 4,632 phage contigs, though the majority of these contigs represent low quality genomes (3581) via scoring by VIBRANT or CheckV (Kieft et al. 2020; Nayfach et al. 2021). As removal of low-quality phage genomes is standard across studies of phage ecology and evolution, and in order to maximize the confidence of microbe-phage HiC interactions in downstream analyses, we retained only 77 phage genomes that we could classify as complete (*n* = 8), high-quality (*n* = 27), or medium-quality (*n* = 42) via CheckV (Nayfach et al. 2021). Recovered phage genomes were dereplicated at 95% ANI and 85% breadth down to 70 genomes (vMAGs), which were used as representative phage genomes for all downstream analyses. Our dereplication strategy reflects known species-equivalent OTU delineations in other bacteriophage communities (Brum et al. 2015; Roux et al. 2016, 2019; Gregory et al. 2019) and resulted in only a 9% reduction in the number of phage genomes, suggesting a large degree of vMAG genomic variation across the three metagenomes. vMAG genome size was highly variable, averaging 28 kb (min 3 kb, max 143 kb) and encoding an average of 35 genes (Table S3).

Next, we sought to identify whether vMAGs cooccurred across multiple honeybee colonies. As each metagenome was derived from a single, distinct colony, cooccurring vMAGs are those that are found at relatively high coverage in more than a single colony. By mapping metagenomic reads from each colony to vMAGs, filtering by genome-wide coverage thresholds, and the number of metagenomic islands (MGIs) (see Methods), we recovered 31 vMAGs that cooccurred in at least 2 colonies, with 6 vMAGs cooccurring in all 3. However, the majority of vMAGs were unique to a single metagenomic sample, with the bulk of vMAGs recovered from metagenomes A and C.

### Metagenomic Islands are Common in Cooccurring Phage Genomes

**Fig. 1.**
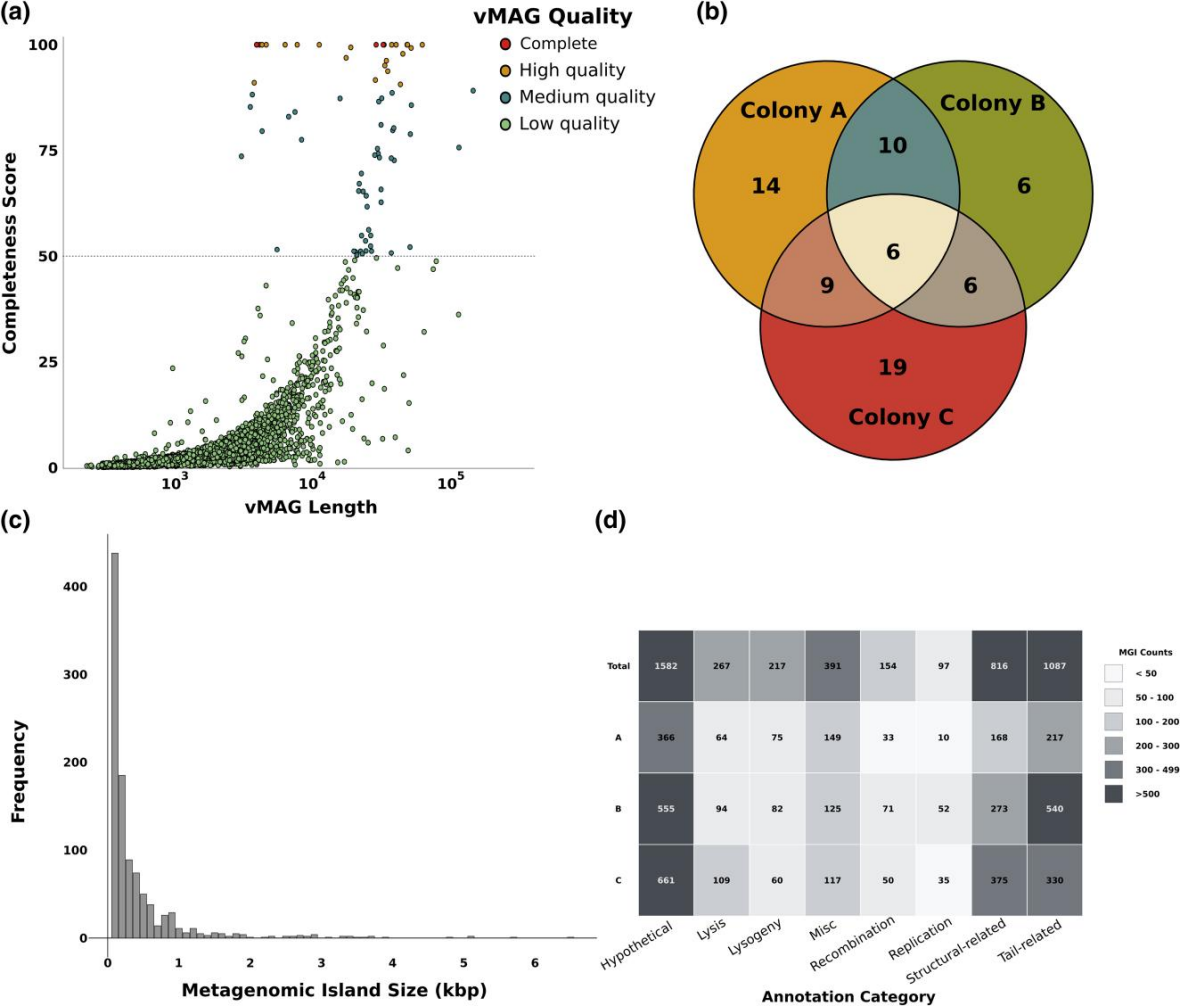
(a) Genome length (x-axis) and checkV quality scores (y-axis) of all recovered vMAGs from the honeybee metagenomic assemblies. Individual points correspond to assembled vMAGs and are colored by quality. (b) Venn diagram showing the number of dereplicated, high-quality vMAGs recovered from each individual honeybee colony. Numbers within circles and overlapping circles correspond to the number of vMAGs that cooccur between any two (or all) colonies. (c) Frequency histogram that shows the count of metagenomic islands (MGIs) that were recovered from cooccurring vMAGs (location of MGIs found within two colonies). MGI size (x-axis) is determined by the number of consecutive MGIs found within a single genome. Larger MGIs have a higher number of consecutive MGI windows. (d) Total count of broadly defined functional gene categories (x-axis) found within each metagenome (y-axis). Counts are determined by the number of observed MGIs that intersect with a phage gene that is grouped within any one functional category.

Microbial genomes—including those of bacteriophages—typically exhibit a high degree of genomic flexibility due to the rapid acquisition and loss of genes. This flux in gene content is driven by a multitude of factors, including selection and drift, and mediated by recombination events (homologous recombination or nonhomologous recombination via interactions with other MGEs) (Hanage 2016). Due to the relatively large number of cooccurring phages in our dataset, we sought to identify regions of phage genomes that were analogous to genomic islands in bacteria (Pašić et al. 2009). By comparing phages that cooccur in >1 metagenomes, we identified MGIs, which are regions of that phage genome that are associated with abnormally low coverage. This low coverage can be driven by deletion events or high genic variation within the phage locus. For this analysis, we focused on the 31 phages found across at least two metagenomic samples. In total, we detected 4,611 individual MGIs with a mean MGI size of 112 bp (ranging from 100 bp to 4,800 bp) (Fig. 1c). Metagenomic islands were detected in all vMAGs that cooccurred in >1 metagenomes, and the summed length of MGIs was associated with an average of 7% of phage genome length. The majority of MGIs were detected in genes with no known function (34.3%). The largest proportion of annotated MGIs fell in genes related to host recognition and entry into the cell. This includes genes related to tail proteins (*n* = 1087) and phage structural proteins, such as capsids and portal proteins (*n* = 816) (Figure 1d). Out of 2,228 recovered genes in cooccurring vMAGs, 692 were associated with at least a single MGI. Individual genes that were most frequently associated with MGIs were genes encoding tail length tape measure proteins (YP_009797605.1) and miscellaneous tail proteins (NP_050160.1). Genes associated with phage recombination (154 total MGIs) and replication (97 total MGIs) were found to be depleted of MGIs in most vMAGs, suggesting potential negative selection to maintain function across individual honeybee colonies.

**Fig. 2.**
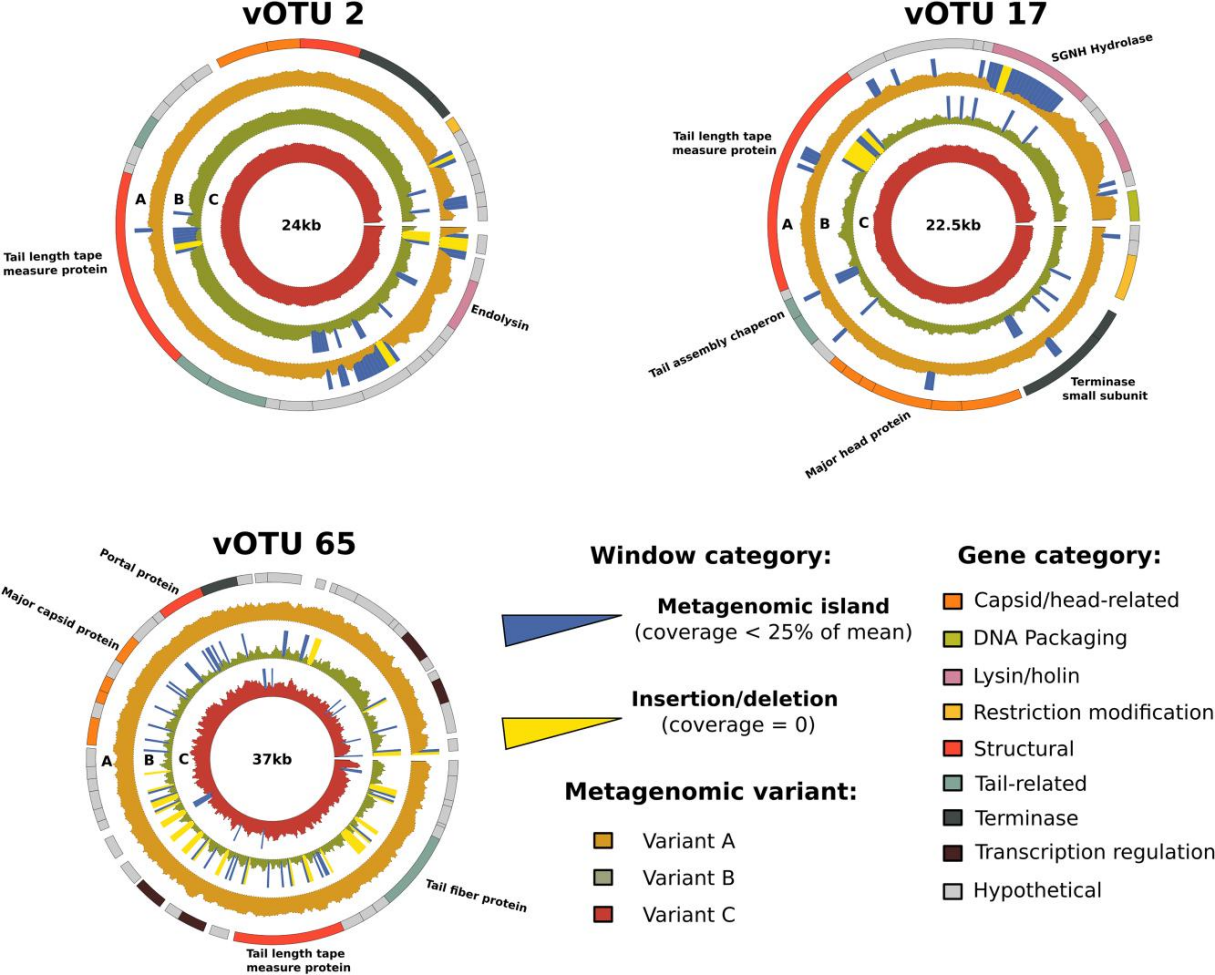
Circos plots of three vMAGs that cooccur in each honeybee colony. The three innermost rings show the genome-wide read coverage within each metagenome. MGIs within a metagenomic variant of the phage are highlighted in blue or yellow. Blue highlights indicate an MGI in which the coverage of the 100-bp window was less than 25% of the genome-wide mean within that metagenome. Yellow highlights indicate an MGI in which the coverage of the 100-bp window was 0. The outermost ring contains the predicted genes within the genome. Gene predictions are colored by function as inferred from DRAM-V.

To more deeply explore MGI variation across vMAGs, we focused our analysis on three well-annotated phage genomes that cooccurred in all three colonies with sufficient coverage depth (10x). The first genome from a 24-kb tailed phage, vOTU 2, was assembled from metagenome C and exhibited relatively few MGIs in both the Metagenome A and B variants. Surprisingly, many of the MGIs in these variants overlapped in genomic positions, with most of the MGIs falling in unannotated genes and a tail length tape measure protein. However, the bulk of vOTU 2 was absent of MGIs across all three variants, suggesting a high degree of genomic stability across colonies (Fig. 2a). Next, we analyzed the 22.5 kb genome, vOTU 17, which was also assembled from Metagenome C. In contrast to vOTU 2, vOTU 17 exhibited profiles of genomic variation across the individual variants (Fig. 2b). Metagenomic islands were recovered from similar positions in both variants within genes related to phage tail length (tail length tape measure protein), tail assembly chaperones, as well as the small subunit of a phage terminase. However, the Metagenome A variant exhibited a fairly small MGI within the phage tail tape measure protein, while the Metagenome B variant was associated with a large MGI of 0 coverage, indicative of an insertion–deletion event. As this protein effectively guides phage tail formation via polymerization with phage tail chaperones, variation in the length of this protein may alter the overall tail length in the assembled virion which could directly impact host range (de Jonge et al. 2019; Yehl et al. 2019). A high number of MGIs were recovered from the vOTU 17 Metagenome A variant within a SGNH hydrolase (Anderson et al. 2022), which is commonly associated with *Acinetobacter*-infecting phages and allows for enzymatic degradation of polysaccharides. Holins and lysins typically exhibit high variability, as their rapid coevolution enables successful phage entry into the cell (Zheng et al. 2008). In contrast, the Metagenome B variant of this gene exhibited a low number of recovered MGIs, suggesting higher conservation in relation to the originally assembled variant. The final genome that we analyzed, the 37-kb vOTU 65, was associated with few MGIs in Metagenome A and C (Fig. 2c). However, the Metagenome B variant was saturated with MGIs, highlighting extensive variability across the length of the genome. Most MGIs of low coverage were scattered through genes related to phage–host interaction, such as the major capsid protein and a portal protein. In contrast, MGIs representing putative indels were distributed fairly evenly among hypothetical genes as well as a gene related to a phage tail fiber protein. The abundance of potential indels in the Metagenome B variant is likely due to gene gain or loss, such as through recombination events with other phages in the honeybee colony.

### The Honeybee Microbiome Contains Phage Populations with Broad Host Range Variation

Having established an estimate of the honeybee phage community within our three colonies and how those phages are partitioned across individual colonies, we next investigated how these phages were distributed among microbial hosts in vivo. Assigning microbial hosts to phage genomes in the absence of isolate-based approaches is challenging, and this approach suffers from intrinsic biases common to all isolate-based methods, such as the recovery of limited microbial diversity as well as biasing against rare taxa and strains. Alternatively, common metagenomic methods for assigning microbe-phage interactions typically rely on matching CRISPR spacers, which are encoded on microbial assemblies, to protospacers that are found on phage genomes. However, this approach suffers from several factors, including that many bacteria do not encode CRISPR systems (Burstein et al. 2016), CRISPR arrays may be misassembled during metagenomic assembly, and the repetitive structures of CRISPR arrays make high-confidence recovery from short read assemblies a challenging task.

We addressed these challenges via HiC metagenomic sequencing of the same three libraries via the Phase Genomics ProxiMeta platform. After filtering HiC reads based on initial read quality, we recovered a total of 345,687,698 Hi-C paired reads (104,694,834, 79,790,796, and 161,202,068 for Metagenomes A, B, and C, respectively). By mapping HiC reads to assembled mMAGs and vMAGs, we can infer potential chromosomal interactions between mMAG and vMAG chromosomes. We estimated noise-to-signal ratios of raw Hi-C contacts using the ratio of intra-mMAG to inter-mMAG contacts (raw noise = 0.0343 ± 0.00417; noise-to-signal ratios were calculated as (Hwang et al. 2023); see Methods for calculations of ratios). In the raw composite HiC network, we recovered 21,068 mMAG x vMAG contacts (Table S4). Metagenomic HiC data exhibit a high degree of noise, represented by spurious contacts between MAGs, and can lead to erroneous associations between phages and their bacterial hosts. We addressed this with a normalization strategy that followed two approaches. First, we used HiCZin to identify spurious vMAG x mMAG contacts based on the number of observed HiC intra-mMAG contacts and the number of unobserved contacts (Du et al. 2022). Next, we calculated the average edge weight of HiC links between *Apilactobacillus kunkeei* and vMAGs from each metagenome to use as the lower bound for which HiC links to include in our analysis. While *A. kunkeei* is widely distributed among honeybee larvae and honeybee queens (Caesar et al. 2024), its distribution and abundance in the worker midgut and hindgut is negligible, and we reasoned that many vMAG x *A. kunkeei* contacts represent putatively spurious and weak interactions. Our results support this reasoning as *A. kunkeei* was the only mMAG that consistently aggregated both the weakest and fewest number of mMAG x vMAG HiC links in both the composite and the individual networks. We note the majority of *A. kunkeei* x vMAG interactions were removed via this method, though some particularly strong interactions remained after filtering. We performed a final filtering step by keeping only vMAGs that were called as present within an individual metagenome as inferred by our phage cooccurrence method. The final composite network saw a 71.7% reduction in the number of vMAG x mMAG interactions, resulting in 5,959 high-confidence mMAG x vMAG interactions (Table S5). Host range distributions across various mMAG hosts are provided as a supplemental figure (Fig. S2).

**Fig. 3.**
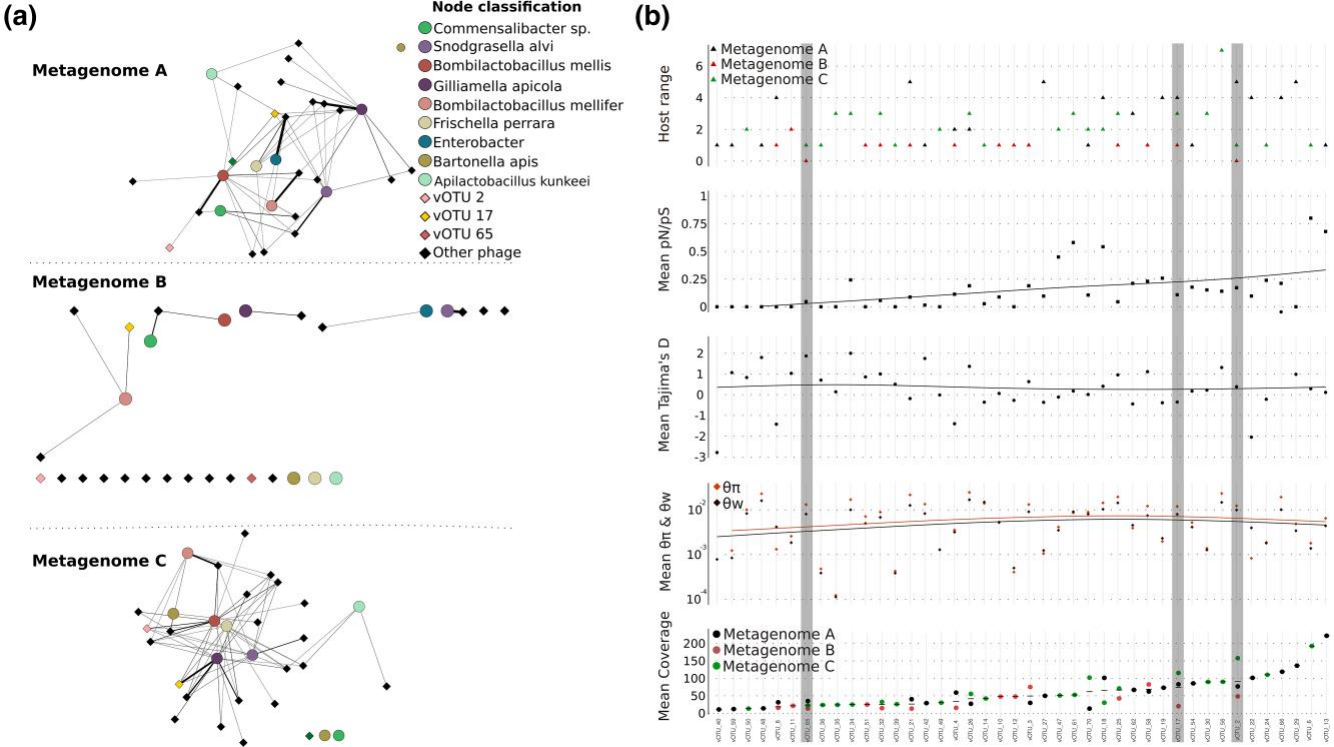
(a) Cytoscape visualization of normalized HiC contacts between mMAGs and vMAGs for each individual metagenome. mMAGs are represented as circles and are colored by genus, while vMAGs are represented as black diamonds. vMAGs that cooccur in all three metagenomes are colored in pink, yellow, and red. Edge width in the network corresponds to the number of contig-to-contig linkages between vMAGs and mMAGs. The darkness of each edge stroke corresponds to the normalized HiC read coverage between any two node pairs. (b) Genomic variation statistics calculated for 42 vMAGs. Only vMAGs where all variation statistics could be calculated are shown. vMAGs were required to have ≥10 × coverage. Gray shaded rows are vMAGs that cooccur in all three metagenomes. The mean metagenomic coverage of each vMAG is represented by a colored dot, with multiple dots corresponding to mean coverage of the same vMAG cooccurring in multiple metagenomes. A small dash between mean coverage corresponds to the mean coverage across all metagenomes. Mean measures of *π* and *θ_w_*, Tajima's D, and genome-wide pN/pS are directly above. These measures are largely stable with increasing coverage. Intra-metagenomic host range (colored triangles) are shown at the top of the figure. The absence of a triangle for any vMAG–metagenome combination indicates that either the vMAG did not cooccur in that metagenome or that the host range within that metagenome is equal to 0.

Within each individual phage HiC network (Fig. 3a), we interpret darker, wider edges between vMAG x mMAG pairs as indicative of the presence of intracellular associations between the pair, though we note that the presence of HiC contacts between any mMAG x MGE pair is not proof of successful in situ infection, nor is it proof of a higher rate of infection within microbial populations. We interpret these signals to describe the distribution of phage interactions within and across microbial hosts and not as bonafide infections. We further note that we interpret the absence of HiC contacts between any mMAG x vMAG pair as resulting from our stringent significance thresholds. We do not interpret absent connections as proof of no interaction. The unnormalized, raw HiC network is provided as a supplemental (Fig. S4; see also Table S5). Overall, we observed that *Snodgrassella*, *Gilliamella*, and *B. mellis* aggregated the highest average number of vMAG x mMAG interactions across all metagenomes. HiC interactions between vMAGs and mMAGs could be dereplicated down to 95 unique links shared between all 9 mMAGs and 42 vMAGs across all 3 metagenomes, suggesting that a large number of vMAGs do not strongly co-localize with any of the recovered mMAGs in our dataset (Fig. S3). Importantly, mMAGs for both *Bifidobacterium* and *Lactobacillus*, which are core bacteria within the worker microbiome, were not recovered with sufficiently low contamination scores to be included in our dataset. Therefore, as common members within the microbiome, these mMAGs are likely hosts for a portion of our recovered vMAGs.

Several vMAGs exhibited broad host ranges, though these interactions varied dramatically in HiC read coverage. We note that observations of broad vMAG host range have been increasingly observed in other systems, including CRISPR-based screens within the honeybee microbiome (Robinson et al. 2025). Overall, we recovered 30 vMAGs with narrow host range (associating with <2 unique hosts within a metagenome) and 28 broad host range vMAGS (25 of these were associated with at least 2 unique genera). vMAGs associated with an average of 2.88 unique mMAGs across all vMAGs exhibited at least 1 significant HiC interaction. However, several vMAGs maintained extremely high HiC coverage with multiple mMAGs. For example, vOTU 66 was associated with *S. alvi*, *B. mellis*, *B. mellifer*, and *B. apis* in Metagenome C and exhibited >50 HiC coverage with both the *Firmicutes* and *α -proteobacteria*. Interactions between vOTU 66, *B. mellifer*, and *B. apis* exhibit higher HiC coverage than the intra-mMAG HiC coverage associated within either *B. mellifer* or *B. apis*. We note that our reported HiC coverage is already normalized by the distribution of intra-mMAG HiC contacts, which suggests that the observed normalized HiC interactions of vOTU 66 are indicative of frequent association. There are many biases intrinsic to the analysis of HiC metagenome data, including bias toward higher coverage and longer contigs (Du et al. 2022). However, linear models showed no significant effect of read depth on the number of mMAG x vMAG HiC linkages (*P* = 0.8729, *R* = 0.001 Fig. S5), while larger vMAGs were associated with fewer interactions (*P* = 1.79 × 10^−12^).

We further examined host range variation within our recovered vMAGs by examining vOTU 2, vOTU 17, and vOTU 65 which cooccur across all three metagenomes. Surprisingly, we found that these vMAGs exhibit vastly different host ranges within each individual metagenome (Fig. 3a). vOTU 2 associated with five, zero, and one host within Metagenomes A, B, and C, respectively. *B. mellis* was the only shared host in Metagenomes A and C. Comparisons of MGIs between these two variants (Fig. 2) suggest that variation in hypothetical genes might drive host range variation in this vMAG, though we note a single MGI within the Metagenome A variant of a tail length tape measure protein. vOTU 2 was associated with four, one, and three unique mMAG hosts in Metagenomes A, B, and C, respectively. The strongest interaction among these variants was with *G. apicola* within Metagenome A, followed by *B. mellifer* and *B. mellis* within Metagenomes B and C, respectively. As reported above, the bulk of the variation in vOTU 2 across all three metagenomes is within genes related to endolysin production and phage tail length. The final cooccurring phage, vOTU 65, exhibited high coverage in all three metagenomes yet was only associated with a single mMAG, *A. kunkeei*, in Metagenome C.

### Phage Host Range Is Associated with Non-synonymous Variants That Are Enriched in Gene Functional Groups

Since we observed that vMAGs were associated with a broad range of hosts across metagenomes, we next investigated whether differences in phage host range exhibited any relationship with measures of genic diversity and adaptive evolution. We operationally define phage host range to include two measures: intra-metagenomic host range and inter-metagenomic host range. We define intra-metagenomic host range as the number of unique vMAG x mMAG interactions within a single metagenome (bound between 0 and 9), while we define inter-metagenomic host range as the number of metagenomes within which a single vMAG cooccurs (bound between one and three). Initially, we calculated common measures of genetic variation across the entire length of the phage genome (Fig. 3b). We observed that measures of diversity, such as nucleotide diversity or Watterson's theta (described further below), varied tremendously between individual vMAG populations and, importantly, did not exhibit any significant relationship with average genome-wide coverage. However, the ratio of non-synonymous to synonymous variants (pN/pS) exhibited a weak, positive relationship with increasing coverage (Table S6).

**Fig. 4.**
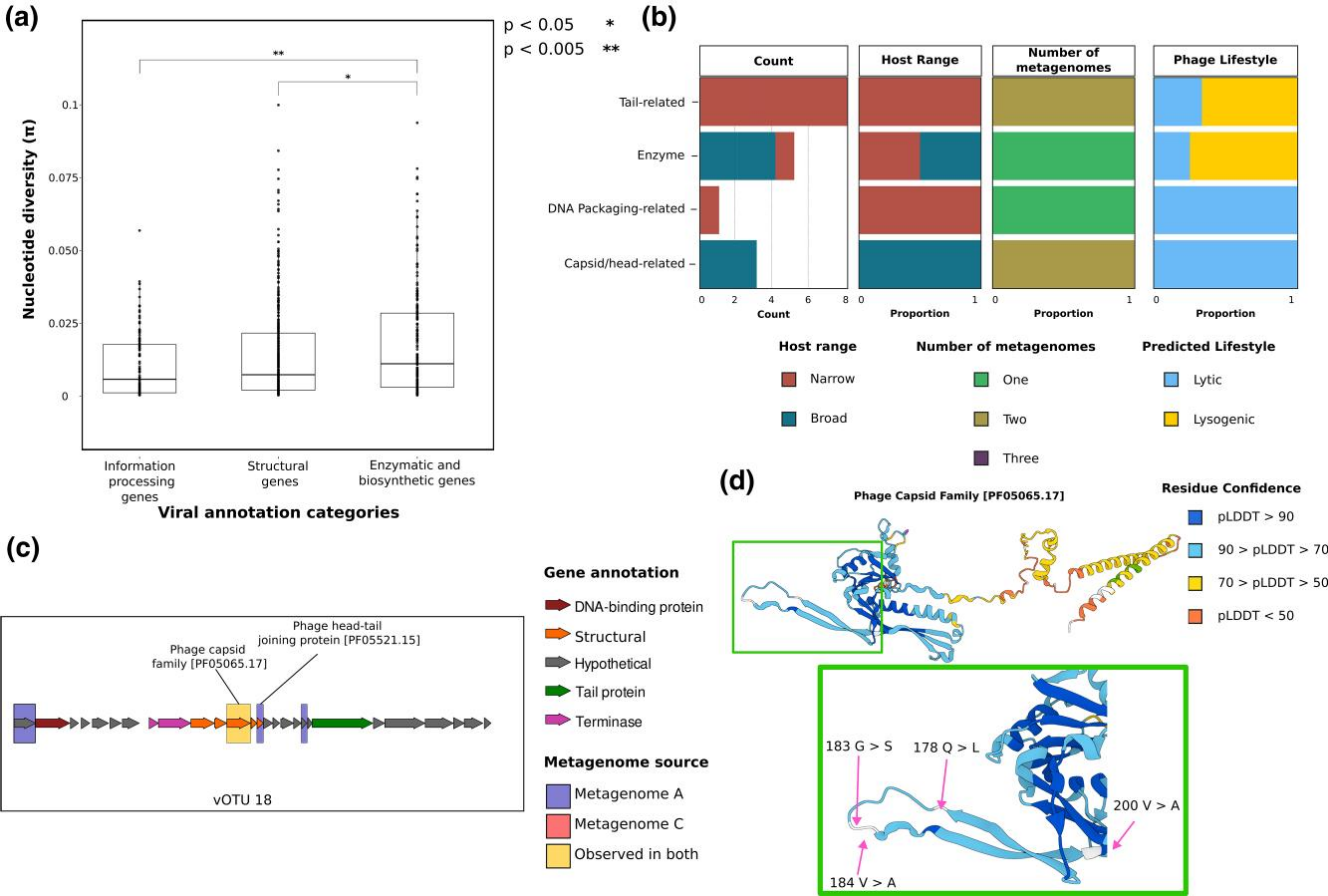
(a) Distributions of gene-wide nucleotide diversity *π* (y-axis) for phage annotation categories (x-axis). Significant differences between annotation categories were tested via pairwise Wilcoxon signed-rank tests. (b) Phage genes with pN/pS ratios ≥1 and their associated phage metadata. Genes are grouped by gene function (y-axis) and their frequency within phage metadata (x-axis). Explored metadata include the count of genes with pN/pS ratios ≥1, the number of unique HiC contacts associated with each vMAG (proxy for host range), the number of metagenomes from which a given vMAG was recovered, and the classification of phage lifestyle based on associated annotations. (c) Gene arrow maps of vOTU 18. Arrows are colored by predicted function. Genes with pN/pS > 1 are highlighted in blue if found only in Metagenome A or in yellow if found in both metagenomes. (d) Alphafold protein prediction of Phage capsid family (PF05065.17) (see Fig. 4c) that shows high similarity to the HK97 fold. Residues are colored by model confidence. Residues associated with non-synonymous variation are highlighted in green, while residues exhibiting high frequency (≥50%) intrapopulation polymorphism are highlighted in white. Amino acid variants are shown for specific residues on the protein's E-loop (green popout), which is known to affect phage protocapsid assembly. Confidence values (pLDDT scores), taken from Alphafold, are provided.

In order to examine relationships between observed phage host range and measures of genetic diversity, we restricted our analysis to phage genes in lieu of full genomic comparisons for three reasons: (i) As seen in Fig. 3b, individual phage species exhibit large variation in estimates of genomic variation, and we opted for gene-based comparisons in order to minimize measures of variation induced by differences in phage species. (ii) Phage genes vary extensively in function, and we sought to understand how different categories of gene function related to measures of genic variation. (iii) We sought to minimize differences in genome quality among individual vMAGs. To explore potential relationships between measures of genic diversity and measures of vMAG host range, we first calculated nucleotide diversity (*π*) for all phage genes in our dataset and compared the distributions of genic nucleotide diversity across four broad functional categories of phage genes (Fig. 4a). Though we coarse-grained gene function by necessity, we found that genes encoding information processing functions harbored the least nucleotide diversity (mean 1.02 × 10^−2^). Comparatively, phage structural genes exhibited large variation in nucleotide diversity (4.22 × 10^−5^ to 9.96 × 10^−2^, mean 1.39 × 10^−2^) and included genes encoding functions related to phage capsid and tail assembly. Enzymatic and biosynthetic genes, as well as unannotated genes (see Fig. S6), were associated with similar measures of nucleotide diversity (mean of 1.749 × 10^−2^ and 1.78 × 10^−2^, respectively) and were significantly different from all other functional categories (pairwise Wilcoxon rank sum test, *P* 0.05 [corrected for multiple comparisons]). Bacteriophage holin genes, responsible for bacterial cell wall degradation, exhibited the highest levels of nucleotide diversity (mean of 7.85 × 10^−2^) within genes related to biosynthetic and enzymatic function, and this may be a consequence of coevolutionary processes between phage and bacterial hosts (Jain and Srivastava 2013; Pollenz et al. 2022). We note that the observed differences in phage-associated genic diversity among different functional groups largely mirrors patterns of diversity observed in genes associated with various bacterial function (Oliveira et al. 2017).

We next sought to explore signatures of adaptive evolution in phage genes due to the differences in the distributions of *π* across categories of gene function. We examined the distribution of pN/pS ratios (the ratio of non-synonymous to synonymous polymorphisms within a population) and identified 52 phage genes potentially undergoing diversifying or adaptive selection (pN/pS > 1); 23 of these genes were observed with pN/pS ratios >2. Eighteen (34.5%) of these genes were associated with some annotated function, while the remaining 65.5% of genes were annotated as hypothetical (see Fig. S6 for hypothetical gene counts). The largest class of annotated genes undergoing putatively diversifying selection were genes associated with phage tail proteins (*n* = 8), such as receptor binding tail proteins (vOTU_40) and phage tail length tape measure proteins (vOTU_51). Genes encoding enzymatic proteins (*n* = 5) and capsid-/head-related proteins (*n* = 4) made up the next two largest classes. Of the enzymatic genes with elevated pN/pS ratios, most predicted functions were related to nucleotide kinases, which may reflect selection for varying rates of DNA synthesis in replicating phage genomes (Tran et al. 2012) or in the regulation of phage gene networks (Guo et al. 2024). Of the capsid-/head-related genes associated with elevated pN/pS ratios, one gene had high similarity to the SPPI bacteriophage *gp16*, which has been shown to be involved in phage head–tail joining and facilitates the driving of phage DNA into host cells (Chaban et al. 2015). Genes exhibiting high pN/pS ratios, and their functional annotations, are provided as a supplemental table (Table S7).

We further investigated the distribution of these genes based on vMAG metadata (Fig. 4b), which included categorized predictions of phage lifestyle, intra-metagenomic host range, and inter-metagenomic host range. For phage lifestyle, we observed that all capsid-/head-related and DNA packaging-related genes undergoing potential diversifying selection were found in vMAGs categorized as lytic, while enzymatic, hypothetical, and tail-related genes were distributed among both lytic and lysogenic vMAGs (see Fig. S6 for full distributions across all annotated gene categories). With respect to intra-metagenomic host range, we found no statistical significance when testing if genes with pN/pS ratios >1 were enriched on vMAGs associated with different intra-metagenomic host range categories (Pearson's chi-squared, *P* = 0.3). However, we observed that genes with pN/pS ratios >1 and encoding tail-related proteins were strictly associated with vMAGs interacting with either zero or one mMAG species (narrow host range vMAGs), while all capsid-/head-related genes that exhibited pN/pS ratios >1 were found on vMAGs that were interacting with ≥2 or more mMAG hosts (broad host range vMAGs). Inter-metagenomic host range found that both diversifying capsid-/head-related genes and tail-related genes were found exclusively on vMAGs with inter-metagenomic host range of 2 (eg cooccurring in two metagenomes).

Based on the observation that genes related to host entry and association and exhibiting pN/pS ratios >1 were found predominantly on vMAGs that cooccurred in two or more metagenomes, we next explored whether targets of diversifying selection were shared between vMAG variants in both metagenomes. We hypothesized that genes of similar function—if not the same gene—might be diversifying since metagenomic variants of the same vMAG may be undergoing similar selective regimes. To explore this, we searched for all vMAGs cooccurring in ≥2 metagenomes and that were associated with at least one gene with pN/pS > 1 in both metagenomic variants. Via this strategy, we recovered a single vMAG (vOTU 18) which was associated with four genes exhibiting pN/pS ratios >1 (Fig. 4c). vOTU 18 cooccurred in both Metagenomes A and C. In the metagenomic A variant, vOTU 18 carried three genes exhibiting high pN/pS ratios and exhibited four unique vMAG x mMAG interactions with *B. mellis*, *B. mellifer*, *Gilliamella*, and *Snodgrassella*. The metagenomic C variant of vOTU 18 associated with a single gene with pN/pS >1 and showed two unique vMAG x mMAG interactions with *B. mellis* and *Enterobacter*. While most genes with elevated pN/pS ratios were unique to each metagenomic variant of vOTU 18, we identified a single gene (phage capsid family [PF05065.17]) that exhibited a pN/pS ratio >1 in both metagenomic variants (Fig. 2c, highlighted in orange). Using Alphafold (Jumper et al. 2021), we found that the protein product of this gene was highly similar (77% identity) to the well-characterized HK97 major capsid protein (or HK97 fold) (Fig. 4d), which is used for protocapsid assembly in HK97 phage.

Since this protein has undergone extensive empirical study (Duda and Teschke 2019), we investigated whether non-synonymous SNVs in either metagenomic variant of vOTU 18 corresponded to potential structural variation in this protein. We first identified the codon positions for each non-synonymous polymorphism in both metagenomic variants of vOTU 18 and then mapped these positions onto the residues of our Alphafold model. We found several non-synonymous variants that were distributed widely across the protein structure, with variant frequencies exceeding 50% in both metagenomic variant vOTU 18. Surprisingly, we identified five non-synonymous variants in the Metagenome C variant of vOTU 18 that were located within the E-loop domain of the protein. At position 178, 62.1% of variants were associated with a glutamine residue, while the remaining variants were associated with a leucine residue. The observed amino acid variants at this residue result in a change in residue polarity, which likely affects the overall structure of the E-domain. HK97 major capsid proteins co-assemble with other capsid proteins to form a phage procapsid structure (Duda and Teschke 2019), and residue changes in this domain have been shown to affect the stability, size, and angle of the procapsid structure (Duda and Teschke 2019; Stone et al. 2019), with consequences pertaining to the size of the phage genome that can be packaged into the capsid.

### Increased Phage Host Range Is Positively Correlated with Measures of Genomic Variation

The distribution of genes with pN/pS ratios ≥1 with respect to variation in phage host range motivated us to more deeply explore relationships between host range and phage genic diversity. Specifically, we hypothesized that both intra-metagenomic and inter-metagenomic phage host range would positively correlate with higher values of allelic diversity (captured by *π*) and the number of SNVs (captured by *θ_w_*) in phage-encoded genes due to a combination of changes in both selection regimes and/or demographic effects. Both *π* and *θ_w_* are estimators of a neutral mutation rate, *θ* = 2Neμ, where *μ* is the population mutation rate and *Ne* is the effective population size. For each gene, we also calculated *Tajima's D* (*D*), which can be understood as the difference between *π* and *θ_w_* (Tajima 1989).

**Fig. 5.**
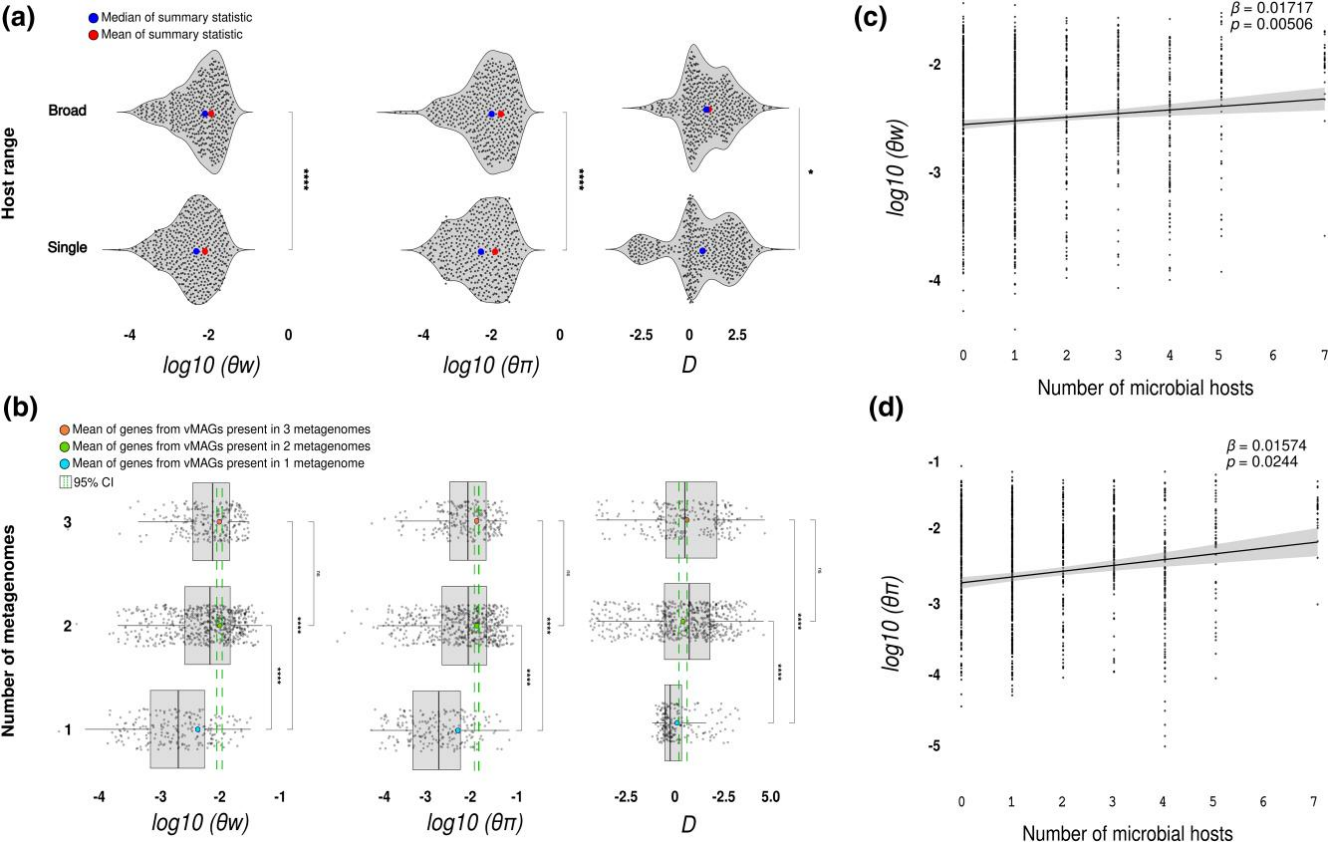
(a) Distributions of genic *θ_w_*, nucleotide diversity, and Tajima's D (x-axis) from vMAGs associated with a single host (bottom distribution) or ≥2 hosts (top distribution). (**** = *P* < 10^−6^, * = *P* < 0.05). (b) Distributions of genic Tajima's D, nucleotide diversity, and *θ_w_* (x-axis) from vMAGs associated with one, two, or three metagenomes (y-axis). Gene-wide variation statistics are significantly higher in vMAGs associated with ≥2 metagenomes (*** = *P* < 10^−16^). (c and d) Correlations between the number of intra-metagenomic mMAG x vMAG filtered HiC contacts (x-axis) and genic measures of *θ_w_* (c) and nucleotide diversity (π) (d). Regression line generated from geom_smooth(method = lm) and shaded gray area represent 95% CI, while *P*-values and beta coefficients were produced from linear mixed models.

Across all vMAGs, we recovered 1,375 genes with which we were able to calculate *π*, *θ_w_*, pN/pS ratios, and *D*. Mean genic values of *π* and *θ_w_* spanned as much as three orders of magnitude (Figs. S7 to S10) and are in line with previous estimates of these statistics using metagenomic data (Sela et al. 2016; Garud and Pollard 2020; N’Guessan et al. 2021), though these published studies measured bacterial populations. Next, we compared differences in intra-metagenomic host range by comparing the distributions of genic *π*, *θ_w_*, and *D* values generated from narrow host range vMAGS (single mMAG host) or broad range vMAGs (≥2 mMAG hosts; Fig. 5a). We found that the broad host range phages exhibited positive shifts in their distributions for all three summary statistics that were calculated *π* (*P* < 1 × 10^−9^); *θ_w_* (*P* < 1 × 10^−7^); *D* (*P* = 0.04199). This trend continued when we compared differences in inter-metagenomic host range. We compared the distributions of genic *π*, *θ_w_*, and *D* values taken from vMAGs associated with one, two, or three metagenomes (Fig. 5b). All three distributions associated with broad inter-metagenomic host range vMAGs (present in ≥2 metagenomes) saw a significant positive shift when compared to vMAGs associated with only a single metagenome (two-tailed Wilcoxon rank sum test, *P* < 1 × 10^−16^). However, genes that were associated with vMAGs that occurred in one, two, or three metagenomes differed significantly in sample size. While the Wilcoxon rank sum test is fairly robust to unequal sample sizes, we addressed this potential source of bias by comparing the mean of each summary statistic associated with vMAGs present in the smallest sample size (vMAGs present in one metagenome) to 95% confidence intervals produced from permutations of the largest distribution (vMAGs present in two metagenomes; see Methods). For each summary statistic, we found that the mean value of genes associated with vMAGs found in a single metagenome was completely excluded from the confidence intervals. The observed positive shift in estimators of *θ* for both intra- and inter-metagenomic host range suggests that phage host range expansion is associated with higher genic and allelic diversity. The positive shift of *D* in both cases is indicative of a higher number of intermediate-frequency polymorphisms, possibly as a consequence of balancing selection or recent population contraction.

Based on our analysis of phage functional genes, we reasoned that the positive shift in summary statistics could be caused by a number of confounding effects, such as annotation or the metagenomic origin of the vMAG. To investigate this further, we performed multiple regression analyses using linear models and linear mixed models to test for the correlation between intra-metagenomic host range and the summary statistics calculated above. In the linear mixed models, gene function, metagenomic source, and the number of metagenomes were treated as random effects. Using this regression approach, we found that intra-metagenomic host range exhibited a significant positive correlation with genic values of *π* ($p<6.4e^{-6}$, β=0.03), $\theta_{w}$ ($p<1.55e^{-5}$, β=0.023), and *D* (p=0.0042, $R^{2}=0.0794$). Analyses using linear mixed models maintained these correlations for $\theta_{w}$ ($p<1e^{-5}$; Fig. 5c) and *π* (p=0.00208; Fig. 5d). This positive correlation remained significant whether or not we included gene length and gene coverage in our models as a control for sequencing bias (π∼intra-metagenomichostrange [p=0.0045]; $\theta_{w}∼\mathrm{intra}\text{-}\mathrm{metagenomic}\;\mathrm{host}\;\mathrm{range}$ [$p<1e^{-4}$]). However, correlations between intra-metagenomic host range and measures of *D* were no longer significant in the generalized model (p=0.37343; see Fig. S11). Though the interaction was still positive, measures of *D* are impacted by gene-specific measures in our dataset, which likely reflect the variation in abundance and genome size of host vMAG chromosomes.

## Discussion

Mobile genetic elements are significant drivers of adaptive evolution among microbial hosts, either through direct coevolutionary interactions or through the acquisition of ecologically important genes. However, microbial x MGE interactions are highly dynamic, and there is a severe paucity of information regarding these interactions in situ. This is especially important within animal host microbiomes, as these dynamics could affect host health via dysbiosis (Forster et al. 2022; Vatanen et al. 2022; Sheahan et al. 2024). Our study directly addresses this knowledge gap through the combination of metagenomic methods and proximity ligation (HiC) with the highly tractable honeybee worker as a model microbiome.

Through the use of HiC sequencing, we show that honeybee-associated phages interact with phylogenetically different microbial hosts in highly structured and extensively coevolved communities. Our observations of the breadth and variation in observed phage host range were surprising. HiC-based methods are notoriously noisy, especially due to biases generated during DNA fragmentation and ligation. These biases include distance-dependent contacts, uneven capture efficiency during amplification, differing GC content among bacteria and viruses, and variation introduced by differences among individual bees. We note that our data are corroborated by several recent studies that use both HiC and CRISPR-based methods to resolve interactions between microbes and their bacteriophages (George and Hug 2023; Hwang et al. 2023; George et al. 2024; Bignaud et al. 2025). Hwang et al. (Hwang et al. 2023) proposed several models to explain the observation of broad taxonomic host range in bacteriophages from a deep sea microbial mat, proposing scenarios such as high density of viral particles among a dense, highly diverse microbial community, the transfer of viral particles and/or genomes among syntrophic microbes, or bonafide host switching or host range expansion in viral populations. While our observations of genic variation in variants of the same viral species with respect to differences to viral host range might support the latter model of viral host switching or range expansion, we stress the need for further experimental work to validate the ability of virions to associate with phylogenetically distant hosts. We emphasize that our observations of vMAG interactions with multiple mMAG hosts are not claims of successful phage infection and reproduction in these hosts. Many of these interactions could result in aborted infections or failure in replication. We also emphasize that our vMAGs represent populations of highly similar phage genomes (5% ANI) which do not possess qualities of a genome generated from a cultured isolate. This limits the ability to resolve fine-scale genetic differences among strains in a metagenome. Genetic tools developed to study phage host range have shown that only a small number of mutations are required in the tail gene gp17 to modify phage host range from *E. coli* to *Klebsiella* or *Yersinia* (Ando et al. 2015). Multiple phage subpopulations within our vMAGs, each persisting on a different microbial host, could potentially explain the observed variation in host range. A larger study, including a combination of closely related isolate genomes, longitudinal sampling, and deeply sequenced HiC metagenomic libraries, might be better able to overcome the significant technical challenges intrinsic to metagenomic HiC data and better resolve the specificity and resolution of phage–strain interactions. In the context of the findings of this study, we hypothesize that broad host phages likely exhibit strain-specific interactions within subpopulations of different bacterial taxa, likely due to recombination within and among phage populations. Further work is necessary to investigate the population structure associated with finer-scale phage-bacterial diversity at levels below the species and/or genus level (eg subspecies or strain diversity). We note that the lack of high-quality assemblies from species belonging to *Lactobacillus* (formerly *Lactobacillus Firm-5* or *Bifidobacterium*) prevents us from describing phage interactions among all core microbial taxa associated with honeybee worker microbiomes.

The observed patterns of molecular evolution based on population genetic summary statistics give further information about viral evolution with respect to host range variation. At least in the viral species that we analyzed, we found that phages that associate with a wider, more diverse set of hosts are associated with a higher accumulation of genetic variation. The significant, positive shift in *Tajima's D* associated with phages cooccurring in multiple metagenomes and in genes found on broad host range phages (Fig. S12) could be explained by the fixation of beneficial mutations on viral genes within some metagenomes but not others, balancing selection via interactions with multiple receptor proteins across bacterial species, recent population contraction in different populations of phage variants, or negative frequency-dependent selection as phage variants oscillate in frequency (Marantos et al. 2022). Regarding targets of selection, the divergent distribution of genes with high pN/pS ratios (>1) with respect to host range suggests a potential evolutionary consequence on phage gene evolution. However, we note that our calculations of *π* and *θ_w_* were made assuming an infinite site mutational model, which assumes that mutations occur only once per site. Due to the high diversity that is observed in viral populations (Gregory et al. 2019), our estimates of *π* and *θ_w_* are likely downwardly biased, though *π* is less affected than *θ_w_* (Tajima 1996). Despite this bias, we interpret the positive correlations between intra-metagenomic host range and measures of nucleotide variation as a consequence of multiple nonexclusive scenarios. Though viral populations are likely very large, genetic drift is likely a major contributor in our system, as expansions in viral host range can drive differences in observed variation due to recent population expansion or bottlenecks. For example, the spread of some viral variant to a new microbial host (resulting in many low-frequency alleles) may cooccur with population contraction of another variant within the viral population. Alternatively, viral variants associating with a broader host range may be exposed to a diversity of selective regimes, and these regimes may not be homogeneous across all variant subpopulations. While more work is needed to support these observations, our current findings may provide clues with which to identify divergent genes in phages undergoing niche partitioning or understanding differences between specialist and generalist phages (Sant et al. 2021).

Interactions among mobile genetic elements and microbial hosts drive many fundamental evolutionary and ecological processes within host microbiomes. Developing a deeper understanding of these interactions is crucial if we are to generate testable hypotheses regarding the response of host-associated microbial communities to perturbation or disturbance, such as in the case of antibiotic usage. Future ecological and evolutionary work will likely benefit from incorporating these observations into predictive models.

## Methods

### Honeybee Management, Metagenomic Sequencing, and HiC Sequencing

Honeybee samples were collected from managed SDI colonies at the Bee Research Facility at the University of Illinois Champaign-Urbana. Single-drone-inseminated colonies were chosen to minimize intra-colony variation among workers (Laidlaw 1987). Each colony was located within the same apiary in order to minimize variability associated due to changes in distance and acquired environmental resources. For each metagenomic sample, a frame was pulled from the colony and visually inspected for the queen. Workers were quickly brushed from the frame and moved to 4C until bees were immobilized. For each individual colony, 15 age-matched workers were removed and dissected, retaining the entire gut of the worker. These guts were pooled and transferred to a sterile Dounce homogenizer. 3 ml of cold PBS was added to each homogenizer and the bee tissue was homogenized on ice. 3 ml of homogenate was then pipetted into three sterile 1.5 ml microcentrifuge tubes and spun down at 500 g for 10 min to pellet host tissue. Supernatant from all three microcentrifuge tubes was transferred to a sterile Falcon tube. 1.5 ml of the pooled supernatant was then transferred to a new microcentrifuge tube and spun down at 5000 g for 10 min. Following this last centrifuging step, the supernatant was removed and the bacterial pellet was flash frozen with liquid nitrogen before being stored at −80C. This process was repeated for all metagenomic samples. We note that the microbial pellet includes both the viral and microbial community and is not specific to the viral component. All samples were sent to Phase Genomics for DNA extraction and the generation of short read and Hi-C proximity ligation libraries via Phase Genomics’ ProxiMeta service. gDNA used for metagenomic sequencing was extracted using zymoBIOMICS DNA Mini Kit. Metagenomic libraries were prepared using a HyperPrep kit (KAPA Biosystems) as referenced in [62]. Hi-C libraries were prepared from a ProxiMeta Hi-C kit. All kits were used according to the manufacturer's instructions. Hi-C libraries were prepared using the restriction enzymes, Sau3AI and MluCI. Both Hi-C and 150 bp short read libraries were sequenced on a single lane of an Illumina NovaSeq.

### mMAG Assembly, Binning, and Annotation

Reads from the metagenomic library were filtered by quality score and trimmed using BBduk (BBMap—Bushnell B.—sourceforge.net/projects/bbmap/). Trimmed reads were then mapped against the honeybee genome (NC_037638.1) before assembly with MEGAHIT (Li et al. 2015) (−min-contig-len 1,000 –k-min 21 –k-max 141 –k-step 12 –merge-level 20,0.95). Microbial metagenomically assembled genomes were binned by combining results from multiple binning software, including Maxbin2 (Wu et al. 2016), MetaBAT2 (Kang et al. 2019), and HiCBin (Du and Sun 2022). These bins were used as input for DASTool (Sieber et al. 2018). Microbial metagenomically assembled genome quality was evaluated using checkM (Parks et al. 2015). We retained only medium-to-high-quality mMAGs (≥50% completeness and ≤10% contamination) from each metagenome. Microbial metagenomically assembled genomes from each metagenome were dereplicated at 97% average nucleotide identity (ANI) using dREP (Olm et al. 2017). Microbial metagenomically assembled genomes were taxonomically assigned using GTDB-Tk (Chaumeil et al. 2020) on KBase (Arkin et al. 2018). Dereplicated mMAGs that were representative of the known phylogenetic diversity within honeybee workers were used for all further analyses. Microbial metagenomically assembled genome genes were predicted using Prokka (Seemann 2014) and annotated using DRAM (Shaffer et al. 2020) and METABOLIC (Zhou et al. 2022) using default databases (DRAM-setup.py prepare_databases –output_dir DRAM_data –skip_uniref).

### vMAG Binning and Annotation

Contigs from each metagenome and ≥3000 bp in length were predicted as phage using VIBRANT v1.2.0 (Kieft et al. 2020). Contigs predicted as phage by VIBRANT were used as input for CheckV (Nayfach et al. 2021). Phage contigs that were classified as medium to high-quality by CheckV were retained for all further analysis. Retained phage contigs were dereplicated with CD-HIT (Fu et al. 2012) at 95% ANI and 85% breadth (cd-hit-est -i $cat_file -o $cat_file_replicate -c 0.95 -*n* 10 -aS 0.85). Dereplicated phage contigs (vMAGs) were taxonomically identified using vConTACT3 (https://bitbucket.org/MAVERICLab/vcontact3/src/master/). Any phages labeled as prophage by VIBRANT or that were dereplicated into a prophage cluster were removed from further analysis. This was done to minimize bias in the HiC network due to stronger prophage–mMAG contacts. vMAGs were annotated using Cenote-Taker2 (Tisza et al. 2021) and DRAM-V and were screened for putative auxiliary metabolic genes (AMGs) using both VIBRANT and DRAM-V.

### HiC-Based Reconstruction of mMAG and MGE Associations

HiC reconstruction of mMAGs and vMAGs closely followed methods described by Hwang et al. (Hwang et al. 2023). Hi-C reads were quality filtered using BBduk and mapped using bwa mem (bwa mem −5SP) [79] against a combined database containing all mMAGs and vMAGs. All contigs in this database were dereplicated a final time using CD-HIT at 95% ANI and 85% breadth to minimize Hi-C reads matching across highly similar contigs. Metagenomic short reads from each individual metagenome were also mapped against this database, and read coverage was calculated using bbduk. Hi-C contact maps were normalized using the unlabeled version of HiCZin (Hamdi et al. 2017) (hiczin.py norm -e Sau3AI -e MluCI). Normalized contacts were further filtered by removing all contacts that exhibited less contact strength (eg HiC paired read coverage) than the average contact strength between *A. kunkeei* and *A. kunkeei*-associated vMAGs. HiC-based contacts between mMAGs and vMAGs were visualized using Cytoscape 3.10.2 (Shannon et al. 2003).

### vMAG Population Genomics and Analyses

Filtered reads from each metagenome were mapped to all conserved single-contig vMAGs using bowtie2 (Tian et al. 2012) in sensitive mode. We calculated the average genome-wide coverage for each vMAG using bedtools (Quinlan and Hall 2010). vMAG contigs, read coverage data, and BAM files were given as input for Anvi’o (Eren et al. 2015). Anvi’o was used to call single nucleotide variants (SNVs) across all phage contigs. Single nucleotide variants were used to calculate microdiversity metrics using custom Python scripts. These scripts are available here: (https://github.com/en-nui/HoneyBeeHiC_public/blob/main/popgen_annotations_summary_statistics_program.py).

### Generation of pN/pS Ratios

The pN/pS ratio is the ratio of intrapopulation non-synonymous (pN) and synonymous (pS) rates. It is similar to estimations of the dN/dS ratio which can be compared across different bacterial strains or species. Analogous to interpretations of dN/dS ratios in protein-coding genes, we can detect putative instances of purifying selection (pN/pS > 1), neutral evolution (pN/pS ≈ 1), and positive selection (pN/pS > 1). pN/pS ratios for each phage protein-coding gene were estimated via Anvi’o (*anvi-get-pn-ps-ratio*) (Eren et al. 2015; Kiefl et al. 2023).

### Calculation of vMAG Nucleotide Diversity

Microdiversity parameters for phage genes were calculated at the gene and genome-wide level. We calculated nucleotide diversity (*π*), Watterson's estimator (*θ_w_*), and *Tajima's D* (Tajima 1989). All statistics were calculated assuming an infinite site mutational model. Nucleotide diversity (*π*), which measures the average number of pairwise nucleotide differences between any two sequences (Hahn 2018), was calculated from SNVs called by Anvi’o via metagenomic reads mapped to phage contigs as follows:


$$\begin{array}{c} \pi =\frac{\sum_{i<j}\;k_{ij}}{n(n-1)/2} \end{array}$$

where $k_{ij}$ equals the number of nucleotide differences between the *i*th and *j*th sequences in the sample and the denominator represents the number of unique comparisons made between *n* sequences (Hahn 2018). *θ_w_*, which is an alternative estimator of *θ*, was calculated as follows where *S* is equal to the total number of segregating sites (or SNVs):


$$\begin{array}{c} \theta_{w}=\frac{S}{a} \end{array}$$

where *a* (a normalizing factor representing the sample size [*n*]) is calculated from


$$\begin{array}{c} a=\overset{n-1}{\underset{i=1}{\sum }}\frac{1}{i} \end{array}$$

Because both *π* and $\theta_{w}$ are estimators of the same parameter *θ*, the expected difference between them should be 0 under the standard neutral model. We estimated the differences between *π* and $\theta_{w}$ via *Tajima's D* (Tajima 1989) as follows:


$$\begin{array}{c} D=\frac{\pi -\theta_{w}}{\sqrt{Var(\pi -\theta_{w})}} \end{array}$$

### Statistical Analyses

All statistical analyses were performed using R v4.4.1. Prior to building linear regressions and linear mixed models, all data were standardized using the Box–Cox transformation in order to ensure the normality of residuals. All linear regression analyses and *t-*tests were built using the *R* function, “summary.lm()” (R Core Team 2021).

### Noise-to-Signal Calculations

Raw noise-to-signal ratios were calculated as Hwang et al. (Hwang et al. 2023):


$$\frac{\mathrm{Number}\;\mathrm{of}\;\mathrm{inter}\text{-}\mathrm{mMAG}\;\mathrm{HiC}\;\mathrm{contacts}}{\mathrm{Number}\;\mathrm{of}\;\mathrm{intra}\text{-}\mathrm{mMAG}\;\mathrm{HiC}\;\mathrm{contacts}}$$

where *inter-mMAG contacts* is equal to the number of HiC read pairs mapping to different mMAGs and the number of *intra-mMAG contacts* is equal to the number of HiC read pairs mapping to the same mMAG.

### Identifying vMAG Metagenomic Variants and MGIs

To identify if a vMAG species was associated with one or more metagenomes, we mapped reads from each metagenome to a database of all recovered, dereplicated vMAGs. Read depth files were generated for each vMAG–metagenome pair. vMAGs that exhibit 10 × genome-wide coverage in a metagenome were considered absent in that metagenome. To account for phage variation due to recombination and HGT, we utilized the frequency of phage MGIs within each metagenome to further determine the presence or absence of a vMAG variant. First, mean genomic coverage was generated for each vMAG–metagenome pair. Next, each pair was split into non-overlapping 100-bp windows. The mean coverage was calculated for each window, and the window was flagged as an MGI if the mean coverage of the window was less than 25% of the mean coverage for the vMAG genome. A vMAG was considered to be in multiple metagenomes if the sum length of the MGIs was less than or equal to the 25% of the total vMAG genome length.

### Effect of Read Depth, Contig Length, and MGE Size on HiC Networks

The effect of read depth and MGE size on the number of mMAG x MGEs HiC linkages was tested via multiple linear regressions:


Y∼Readdepth

Y∼MGEsize

Y∼Contiglength

where Y represents either the number of HiC contacts or the unique number of microbial hosts for vMAGs. The significance of these multiple linear regressions were evaluated with the *t*-test of the R function *summary.lm()*. The *p.adjust()* function of the R package “stats” was used to adjust the linear regression *P*-values using FDR correction.

### Variation Across Annotation Categories and Presence in Multiple Metagenomes

Measures of genomic variation (*π*, *θ_w_*, and Tajima's *D*) were calculated separately for each vMAG-associated gene and binned into one of four major annotation categories (information processing, structural genes, enzymatic and biosynthetic genes, and hypothetical genes). These categories were built from KEGG BRITE Pathway categories. Differences in genomic variation among the four functional groups were tested via pairwise Wilcoxon rank sum tests. *P*-values were corrected for multiple tests as above. This procedure was applied to test for differences in genic variation among vMAGs associated with one, two, or three metagenomes. Plots were generated with ggplot2 (Wickham 2016).

### Identifying Phage Lifestyle Categories

Phage contigs were classified as either lysogenic or lytic based on the presence of specific annotations. Our approach is similar to that developed in (Mavrich and Hatfull 2017). The predefined list of keywords was associated with lysogenic phages and mu-like phages. Phages associated with any of these keywords were labeled as putatively temperate, and the absence of any keyword resulted in vMAGs being labeled as lytic. The list of keywords are as follows: *parA*, *parB*, *parS*, *repressor*, *recA*, *recB*, *recE*, *recT*, *rusA*, *ruvC*, *resolvase*, *integrase*, *excision*, *excisionase*, *recombinase*, *immunity repressor*, *excise*, *mu-like*, *repress*.

### Effect of Host Range on Phage Gene Sequence Variation

To investigate the relationship between phage host range and measures of genic variation, we used linear mixed models through the R package, lme4 (Bates et al. 2015). We opted for linear mixed models to better incorporate random effects. Random effects are grouping factors that explain random variance of the relationship between the response variable and fixed effects across a number of different groups (N’Guessan et al. 2021). Further, our data violates assumption of independence due to the presence of vMAGs in two or more metagenomes as well as the possibility of HGT-mediated acquisition of near-identical genes across vMAGs. Linear mixed models are robust to these violations. Before regression modeling, we removed all vMAGs with 0 vMAG x mMAG interactions across all metagenomes. Next, data were normalized using the Box–Cox transformation to ensure the residual normality. The regression models are presented as below:


$$\theta_{\pi }^{\;}∼\mathrm{Phage}\;\mathrm{host}\;\mathrm{range}+\mathrm{Annotation}+\mathrm{Metagenome}\;\mathrm{source}+\mathrm{Number}\;\mathrm{of}\;\mathrm{metagenomes}$$

$$\theta_{w}^{\;}∼\mathrm{Phage}\;\mathrm{host}\;\mathrm{range}+\mathrm{Annotation}+\mathrm{Metagenome}\;\mathrm{source}+\mathrm{Number}\;\mathrm{of}\;\mathrm{metagenomes}$$

$$\mathrm{Tajim}a^{\prime }s\;D^{\;}∼\mathrm{Viral}\;\mathrm{host}\;\mathrm{range}+\mathrm{Annotation}+\mathrm{Metagenome}\;\mathrm{source}+\mathrm{Number}\;\mathrm{of}\;\mathrm{metagenomes}$$

where θπ, *θ_w_*, and *Tajima's D* are values calculated for each vMAG-associated gene (*n* = 1375). Intra-metagenomic host range was defined as the number of unique intra-metagenomic mMAG hosts. Annotation, metagenome source, and number of metagenomes were included as random effects to account for sequence variation between these groups. Asterisked values incorporate gene coverage and length into the model as random effects. *P*-values and effect coefficients for each regression model were calculated via the R package “lmerTest.” Tests of differences in the measures of genic variation associated with differences in inter-metagenomic host range (defined as the number of metagenomes with the same cooccurring phage species) were done via two-tailed Wilcoxon rank sum tests on untransformed data. To account for differences in sample size associated with each group, *N* number of genes were sampled from the larger distributions, where *N* is equal to the number of genes in the smallest distribution (vMAG genes present in a single metagenome). This process was repeated 1,000 times to produce a 95% confidence interval around the mean of the larger distributions. *P-values* were calculated for both the Wilcoxon rank sum test and from the frequency with which the mean of the single-metagenome vMAG genes was found within this 95% confidence interval. All plots were generated with ggplot2.

## Supplementary Material

evag152_Supplementary_Data

## Data Availability

Raw metagenomic and HiC reads are available from NCBI under BioProject PRJNA1206451. Links to many of the Python workflows, as well as greater detail regarding how our analyses were done, can be found here: https://github.com/en-nui/HoneyBeeHiC_public.
