# Supplementary figures and images for: Host Range Breadth Correlates with Genic Diversity in Honeybee Phages
Source: Genome Biol Evol. 2026 Jul 3;18(7):evag152. doi: 10.1093/gbe/evag152 (PMC13331135; doi:10.1093/gbe/evag152)

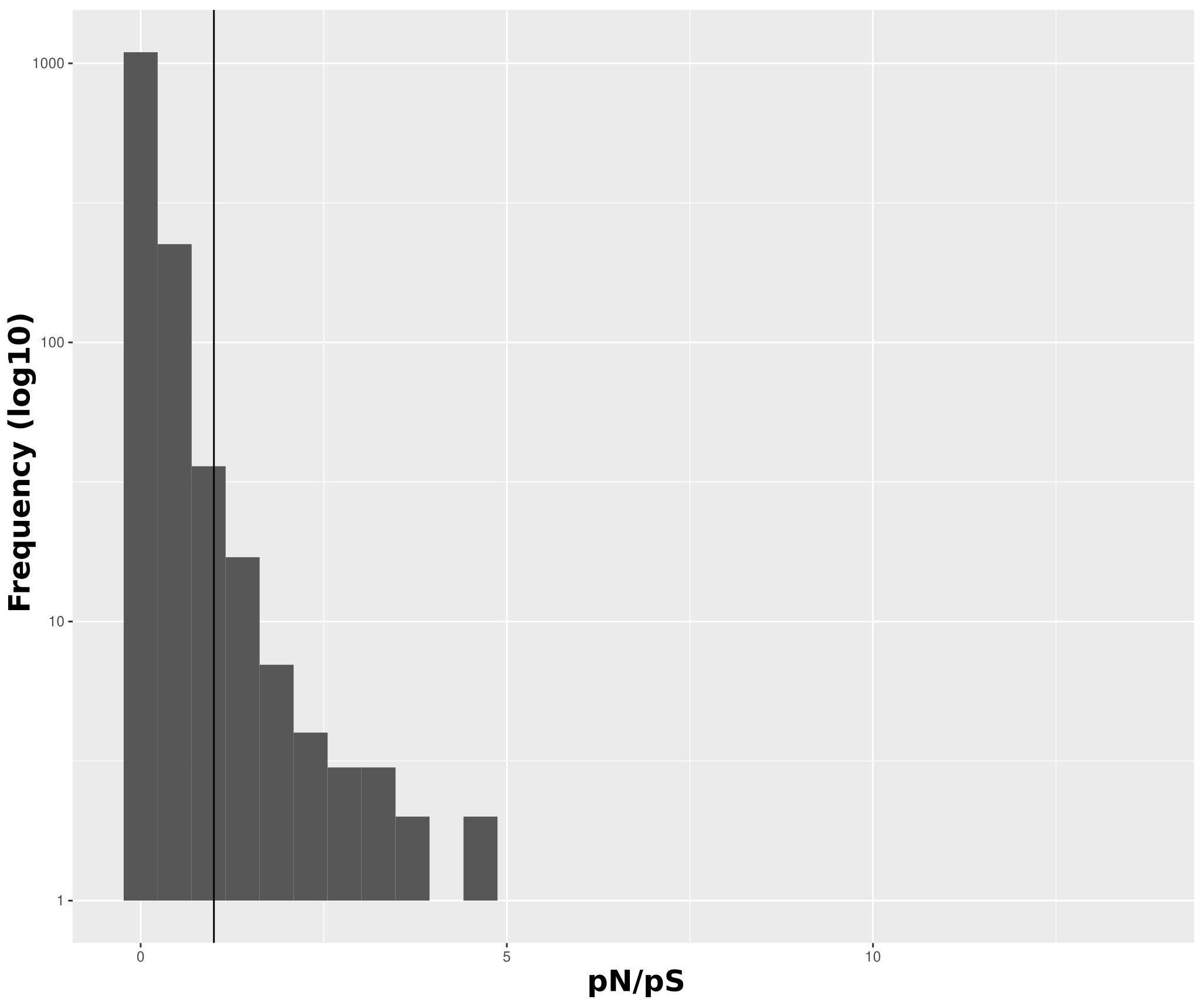

Supplement: evag152_Supplementary_Data [file evag152_supplementary_data.zip › fig10.png]

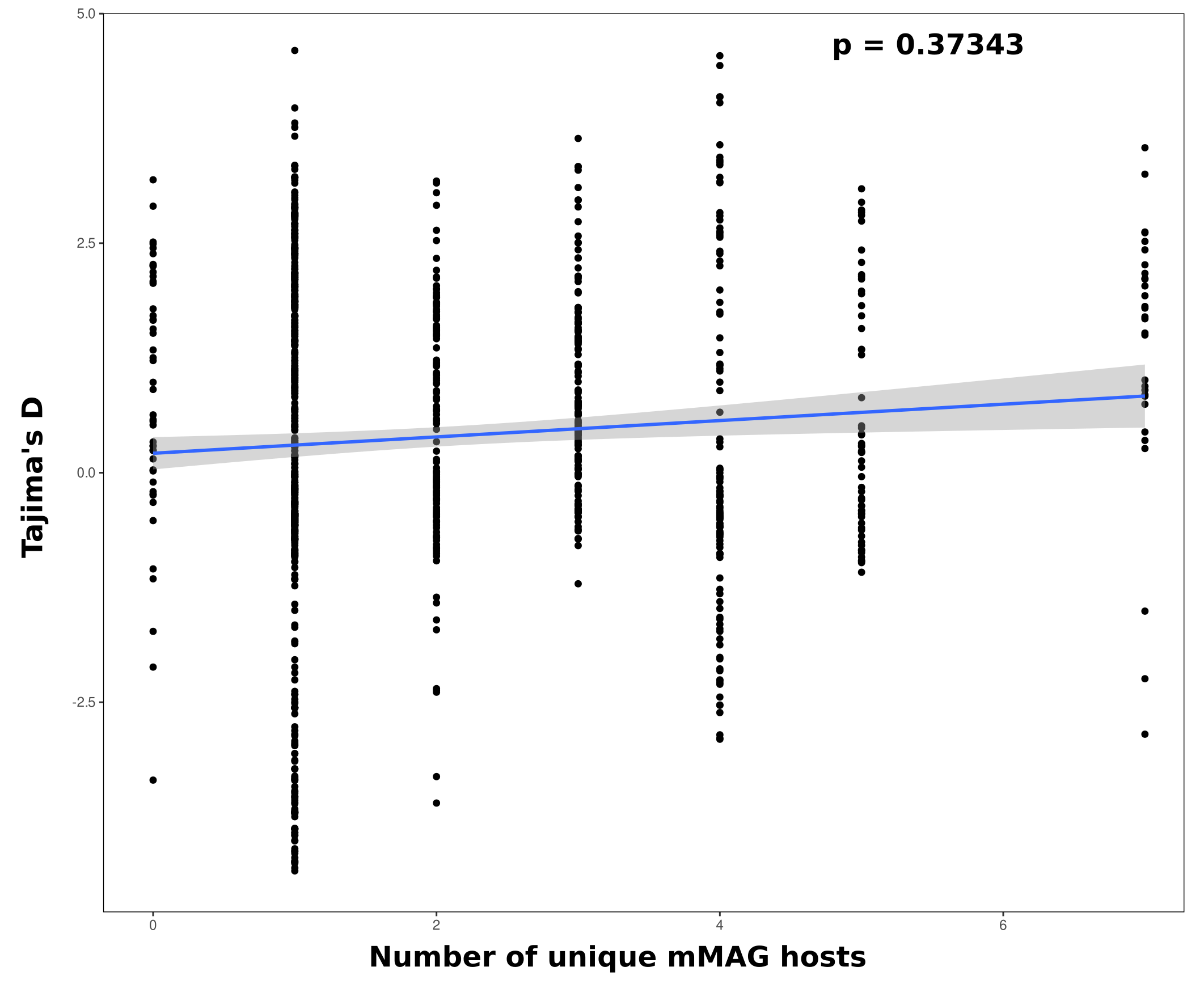

Supplement: evag152_Supplementary_Data [file evag152_supplementary_data.zip › fig11.png]

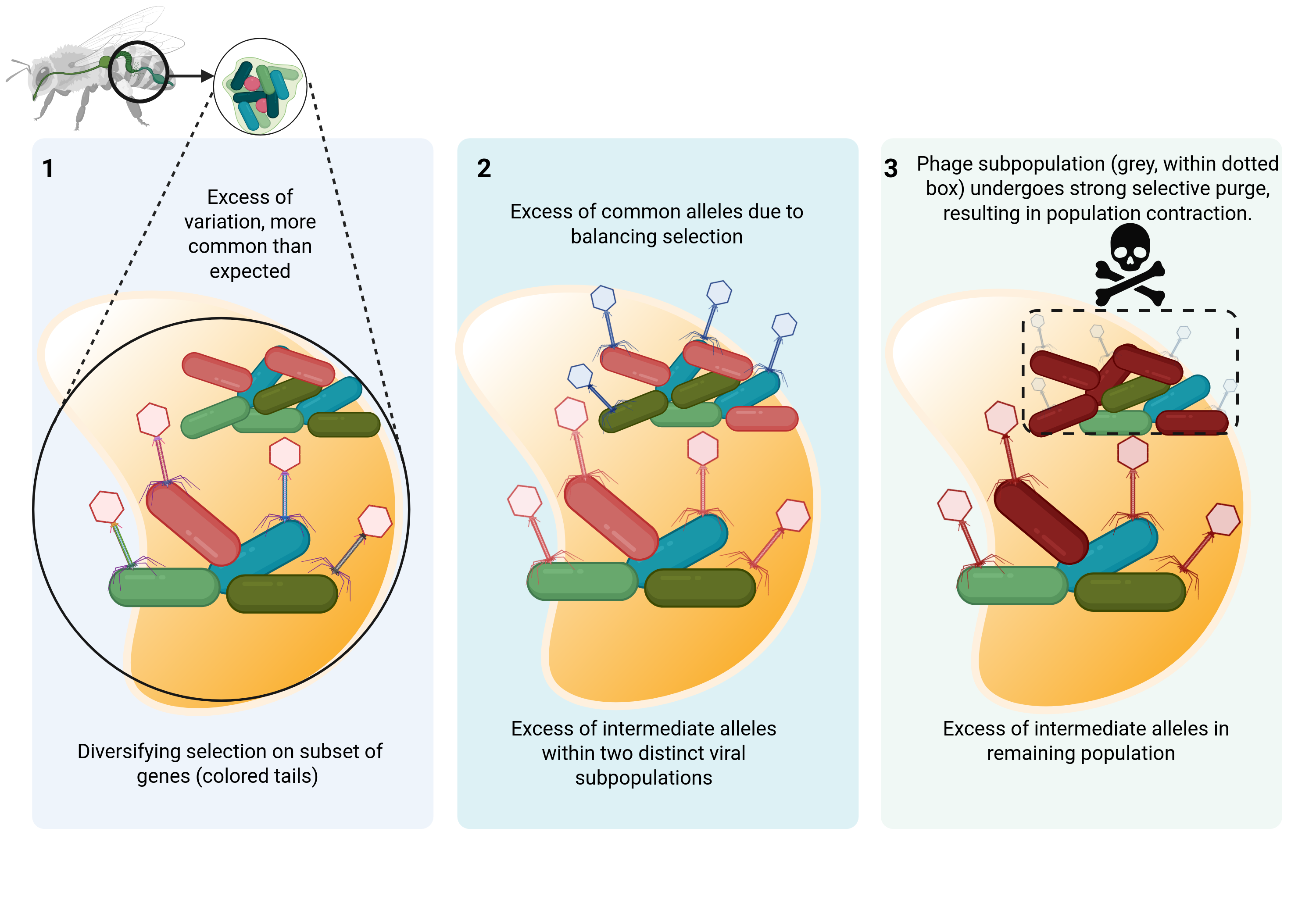

Supplement: evag152_Supplementary_Data [file evag152_supplementary_data.zip › fig12.png]

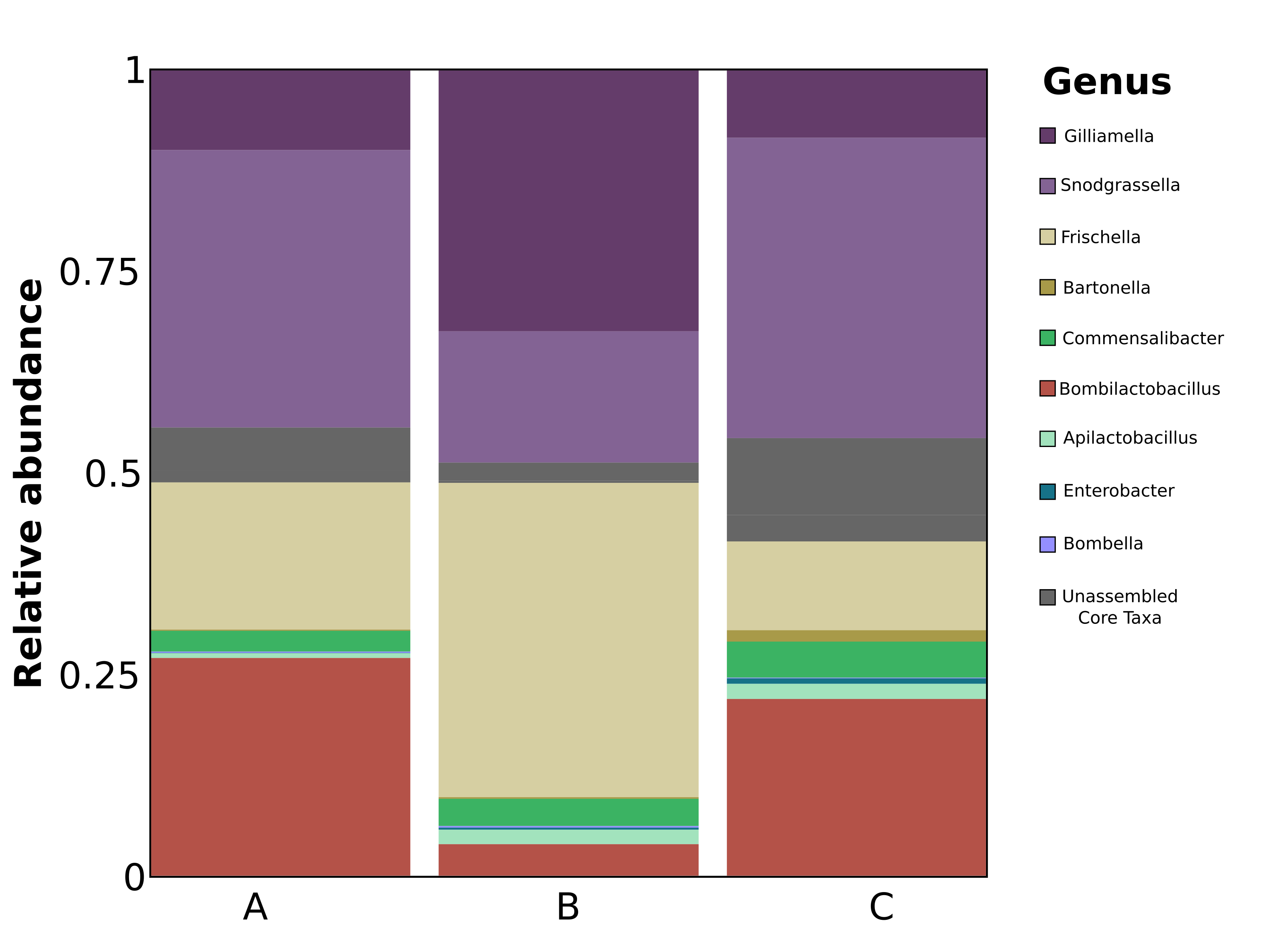

Supplement: evag152_Supplementary_Data [file evag152_supplementary_data.zip › fig13.png]

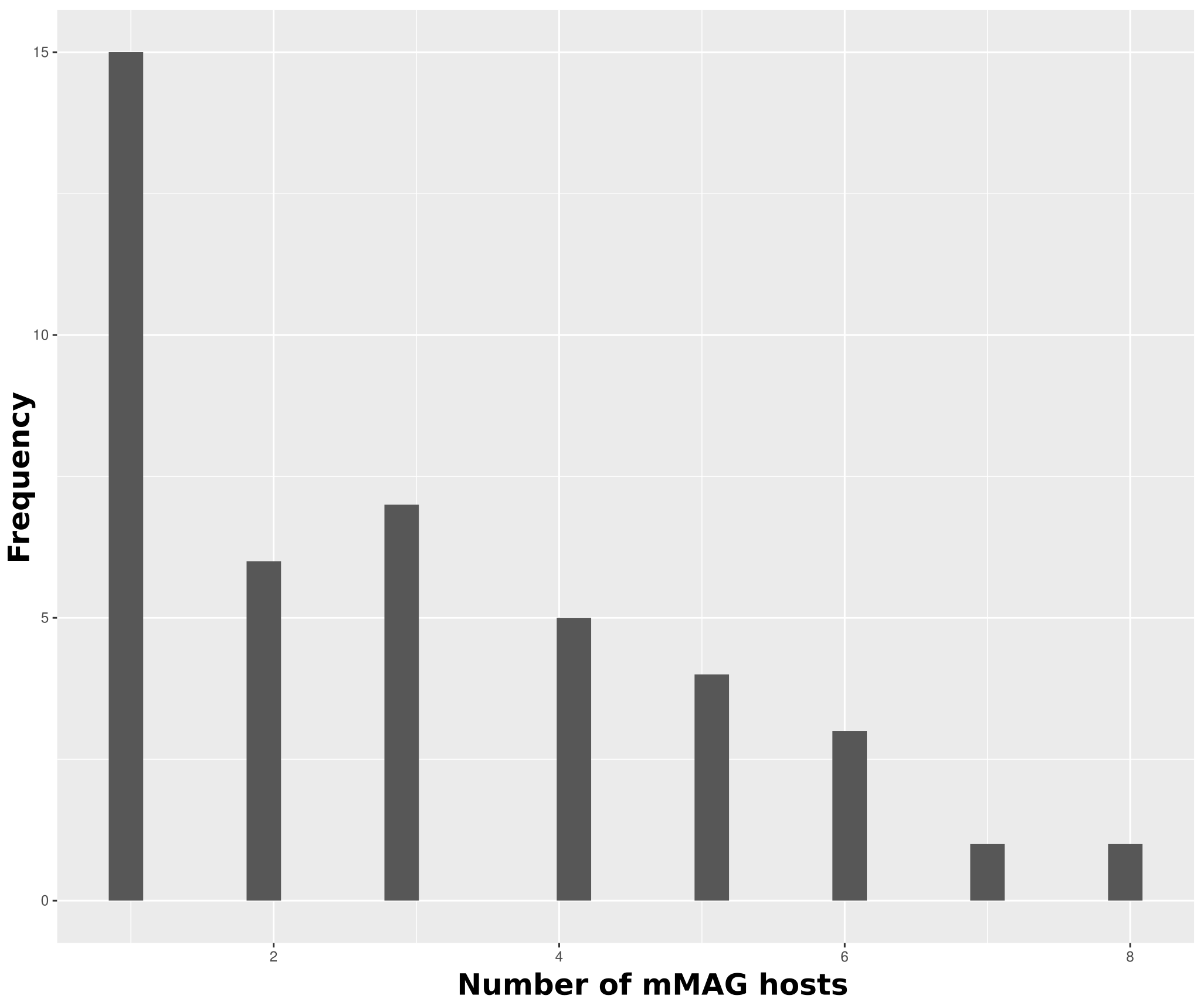

Supplement: evag152_Supplementary_Data [file evag152_supplementary_data.zip › fig2.png]

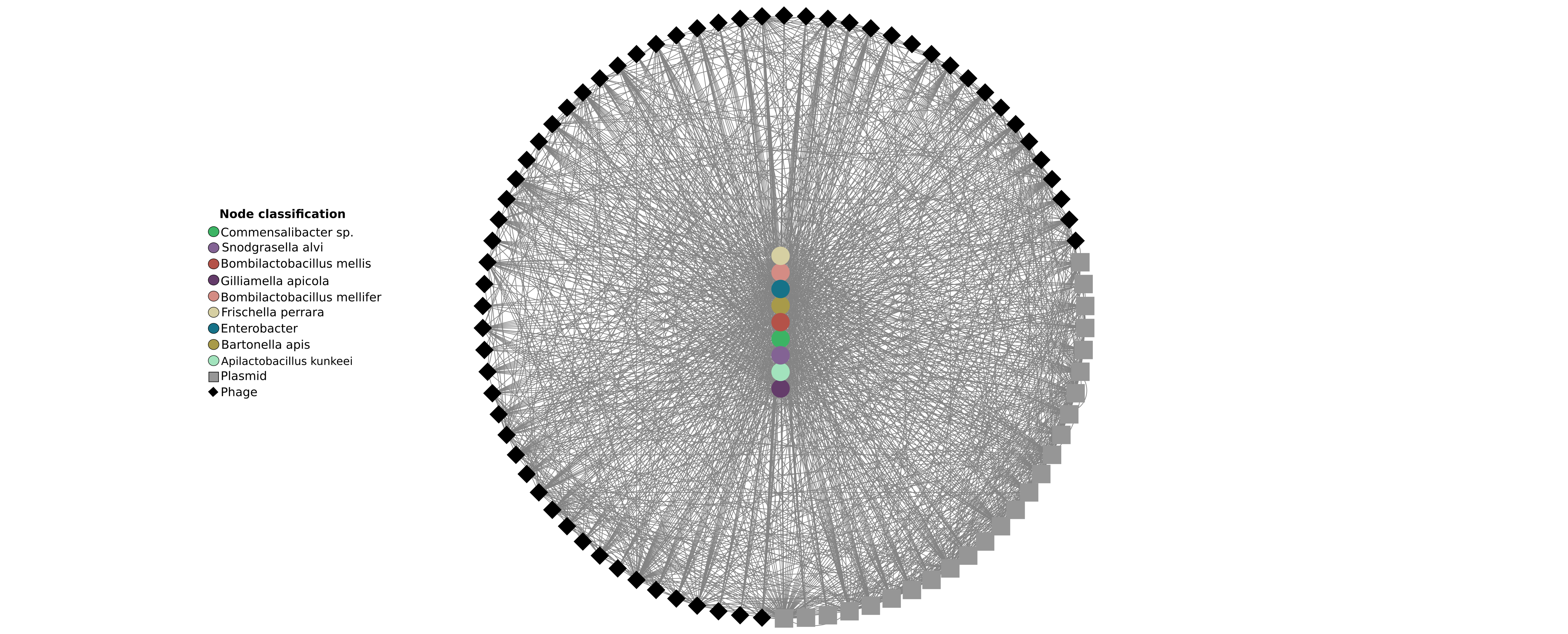

Supplement: evag152_Supplementary_Data [file evag152_supplementary_data.zip › fig3.png]

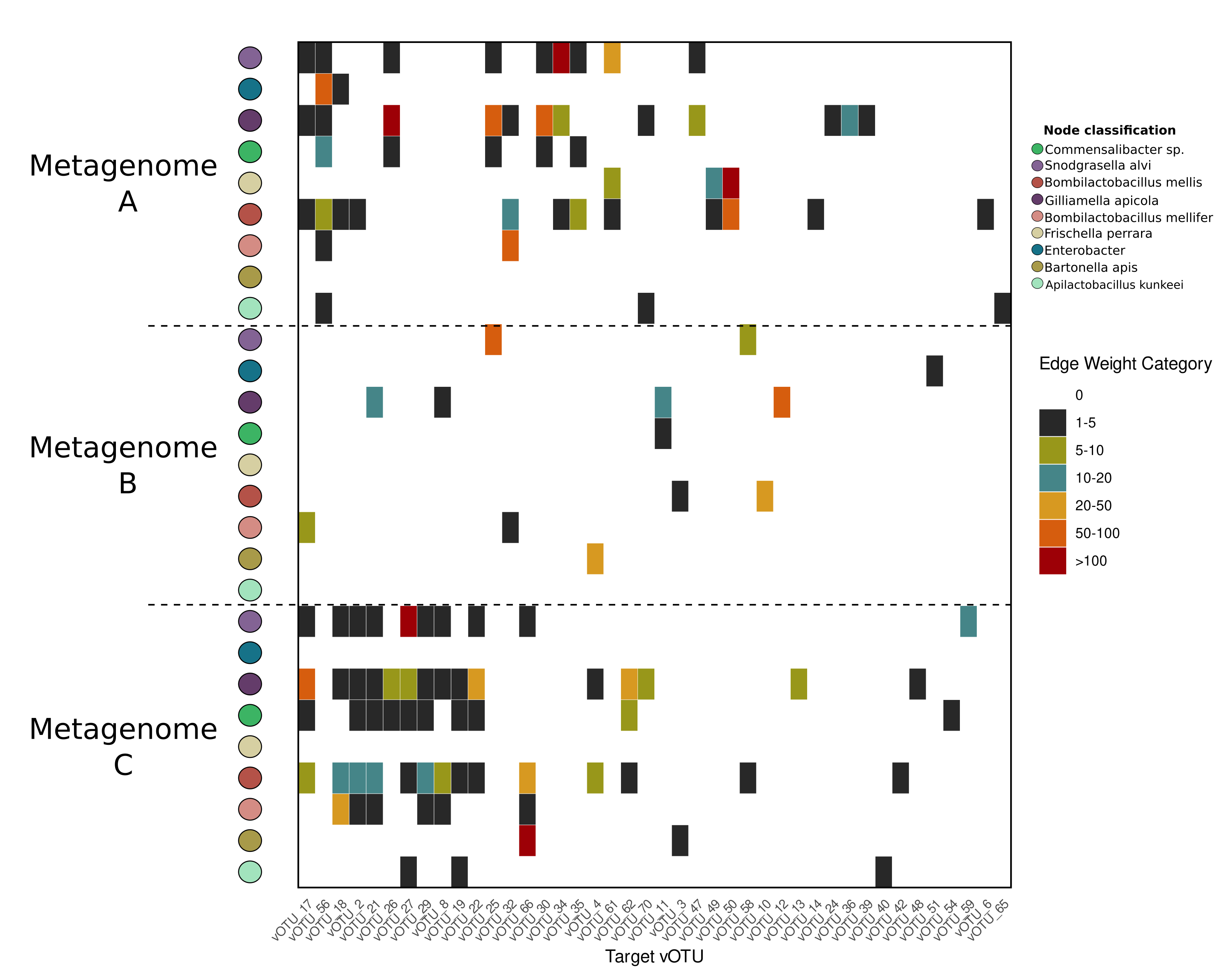

Supplement: evag152_Supplementary_Data [file evag152_supplementary_data.zip › fig4.png]

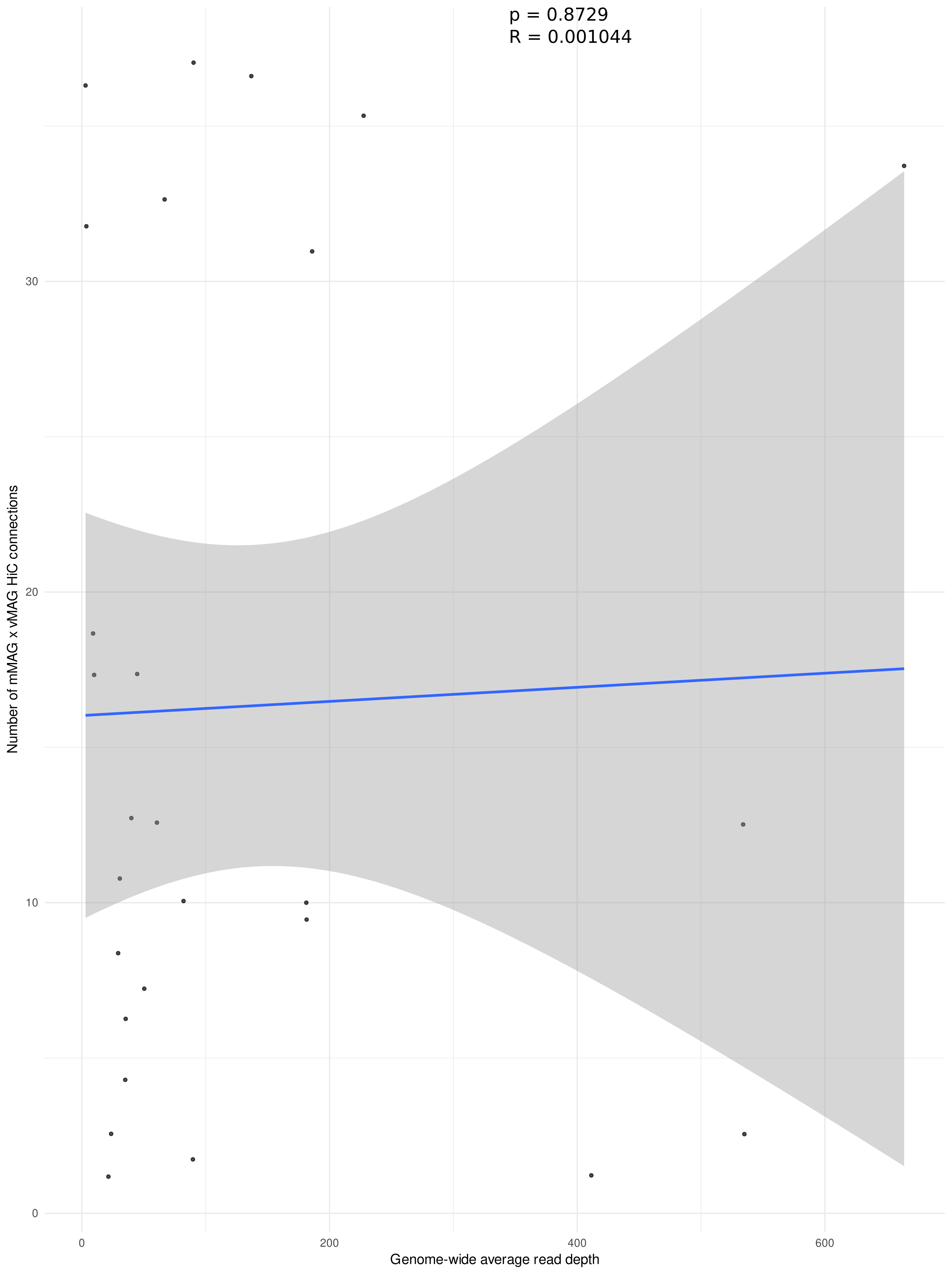

Supplement: evag152_Supplementary_Data [file evag152_supplementary_data.zip › fig5.png]

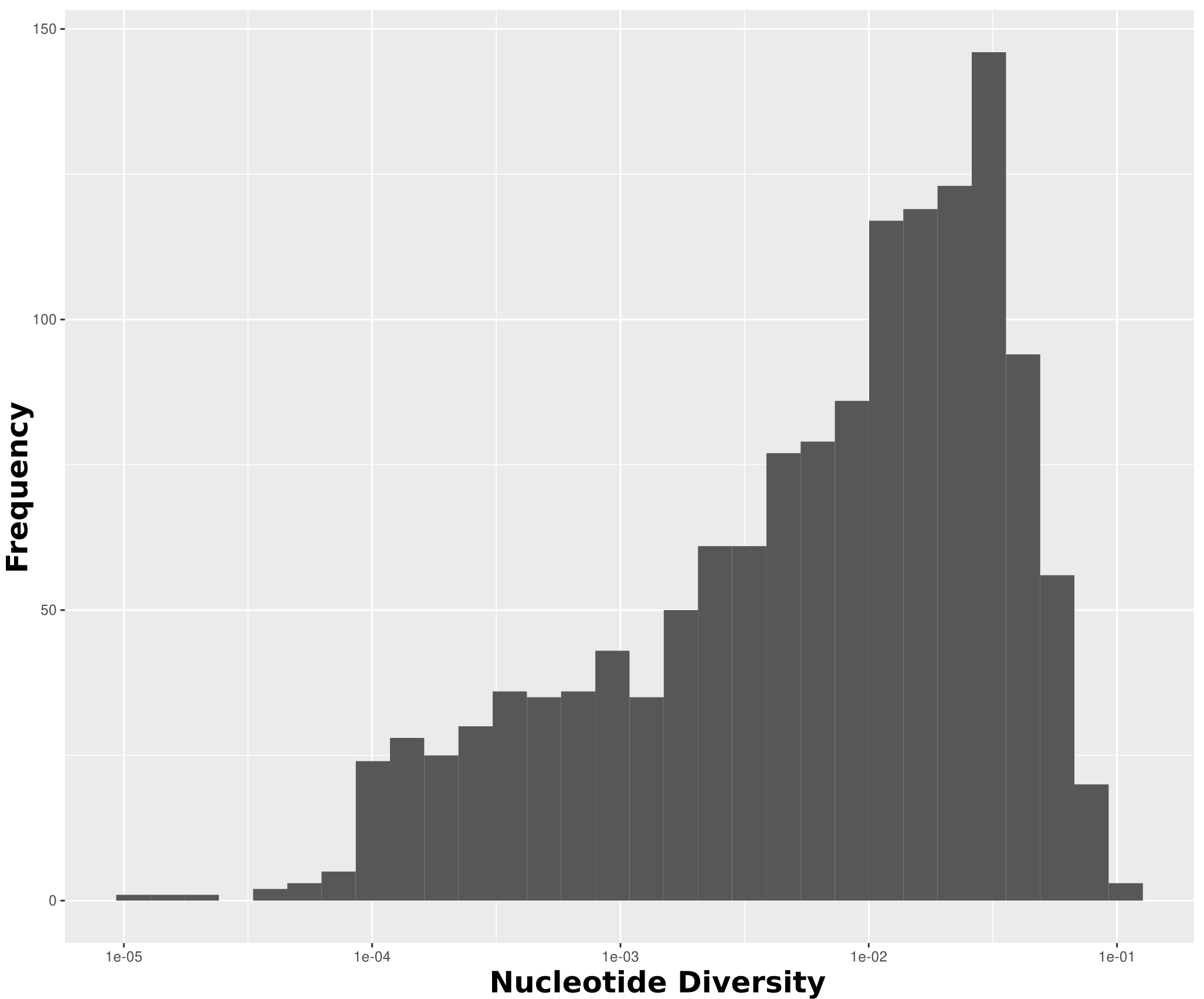

Supplement: evag152_Supplementary_Data [file evag152_supplementary_data.zip › fig7.png]

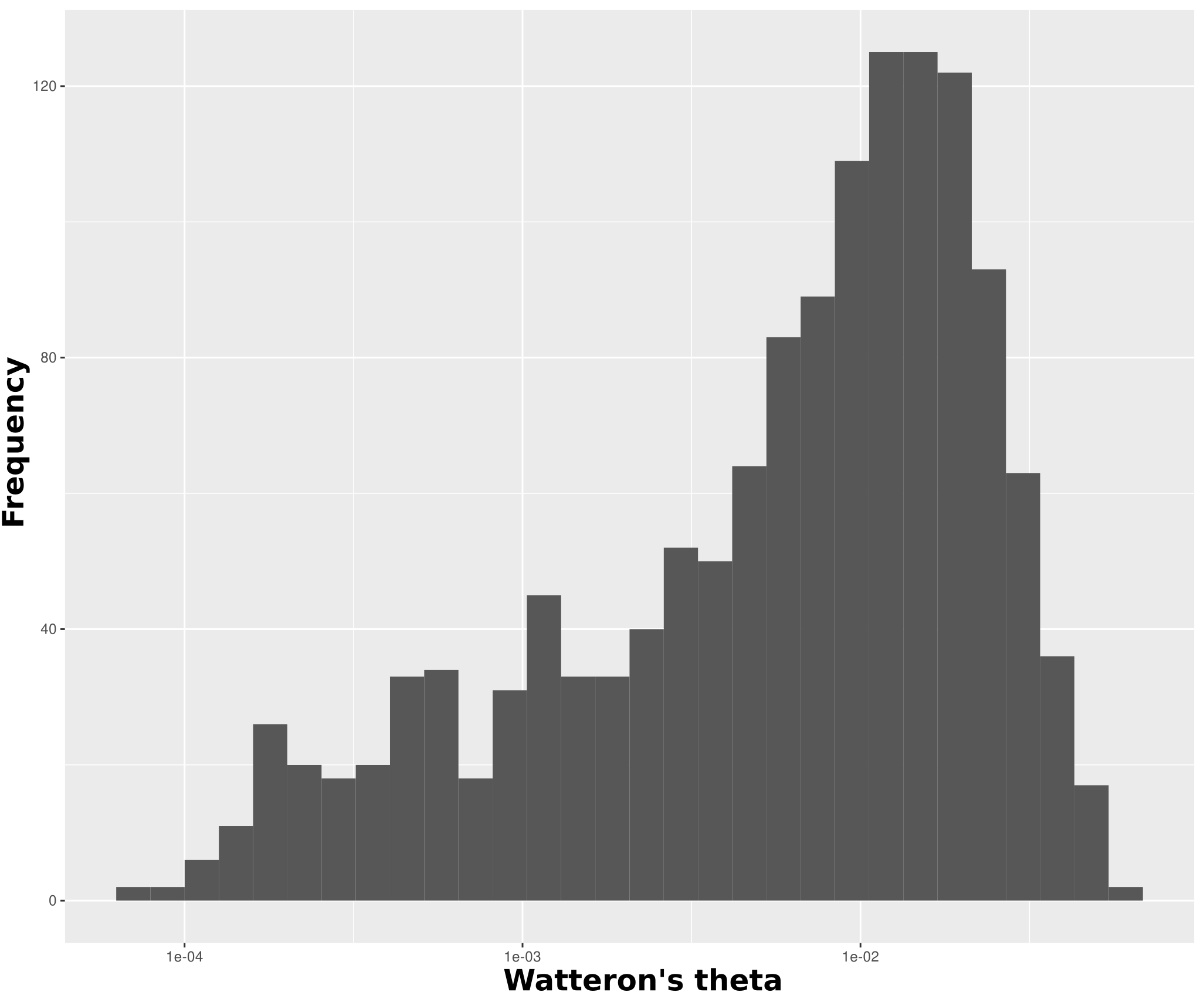

Supplement: evag152_Supplementary_Data [file evag152_supplementary_data.zip › fig8.png]

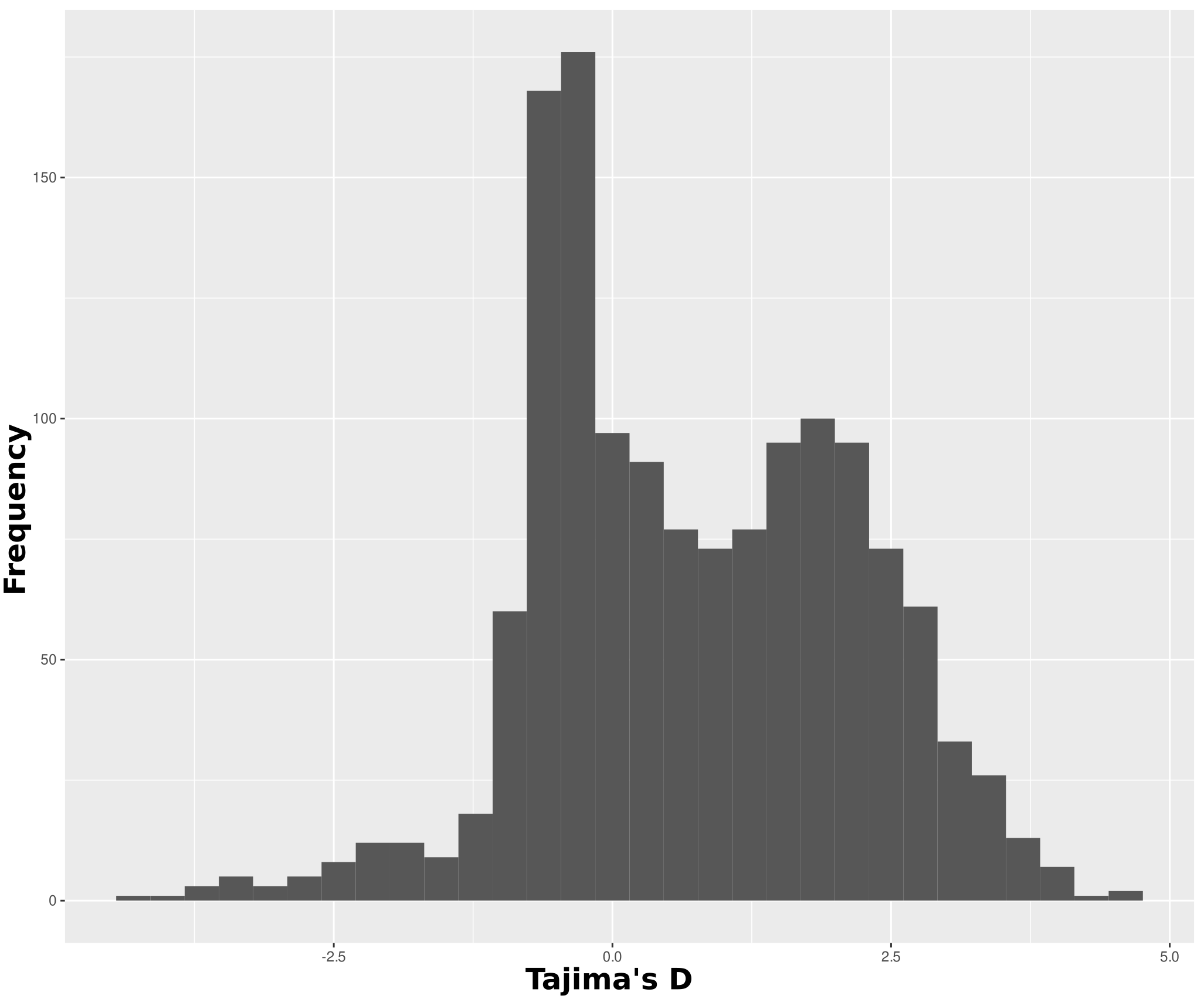

Supplement: evag152_Supplementary_Data [file evag152_supplementary_data.zip › fig9.png]

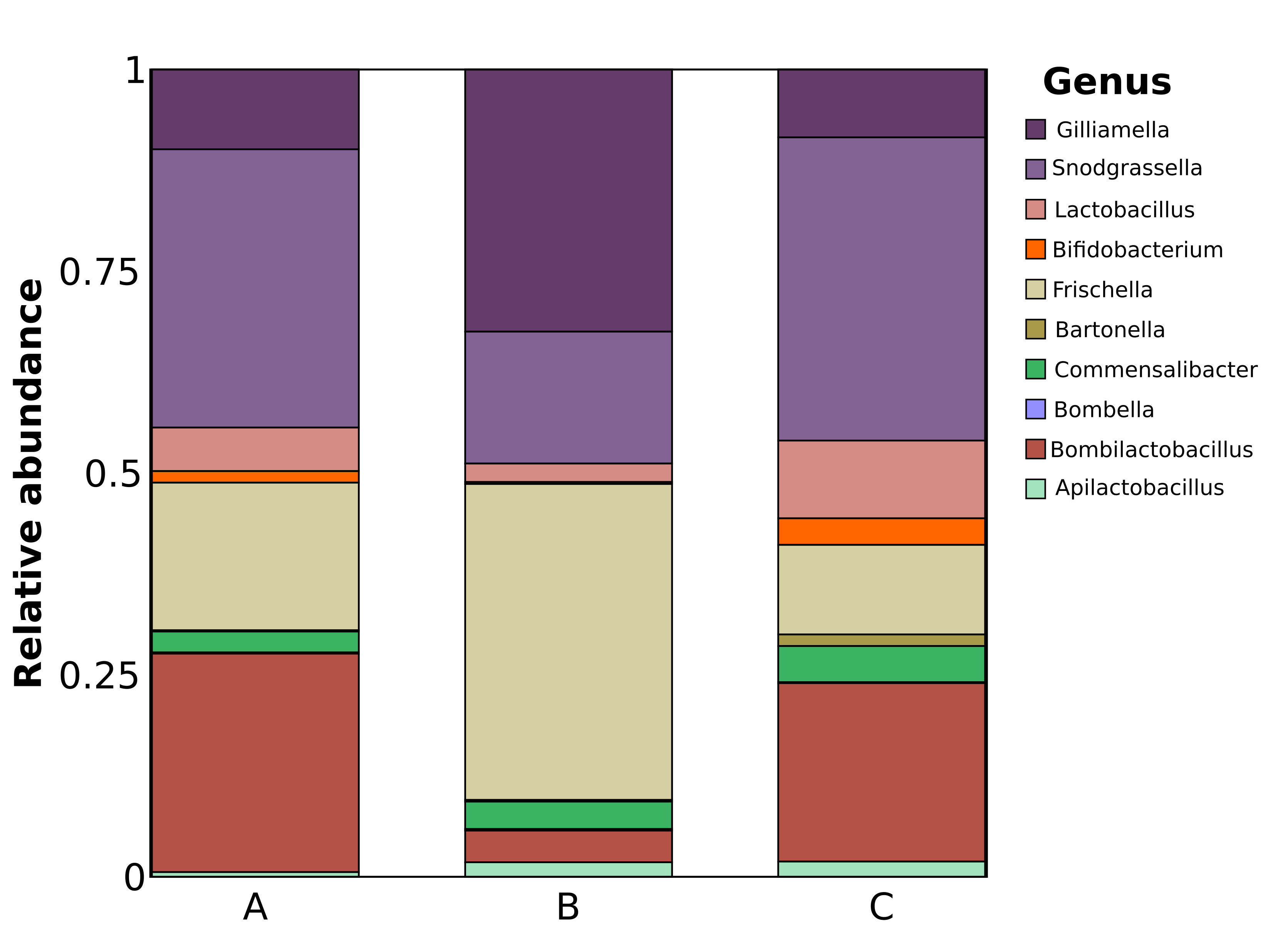

Supplement: evag152_Supplementary_Data [file evag152_supplementary_data.zip › SuppFig1.png]
